# Dual-dynamic-bond cross-linked injectable hydrogel of multifunction for intervertebral disc degeneration therapy

**DOI:** 10.1186/s12951-022-01633-0

**Published:** 2022-10-01

**Authors:** Linjun Yang, Congcong Yu, Xuhui Fan, Tianni Zeng, Wentao Yang, Jiechao Xia, Jianle Wang, Litao Yao, Chuan Hu, Yang Jin, Yutao Zhu, Jiaxin Chen, Zhijun Hu

**Affiliations:** 1 Department of Orthopaedic Surgery, Sir Run Run Shaw Hospital, Key Laboratory of Musculoskeletal System Degeneration, Regeneration Translational Research of Zhejiang Province, Zhejiang University School of Medicine, 3 East Qing Chun Road, Hangzhou, 310002 People’s Republic of China; 2 Department of Radiology, Shanghai General Hospital, Shanghai Jiao Tong University School of Medicine, 100 Haining Road, Shanghai, 200080 People’s Republic of China; 3 Department of Oncology, Hangzhou TCM Hospital Affiliated to Zhejiang Chinese Medical University, 453 Tiyuchang Road, Hangzhou, 310007 People’s Republic of China; 4 Department of Dentistry, Sir Run Run Shaw Hospital, School of Medicine, Zhejiang University, 3 East Qing Chun Road, Hangzhou, 310002 People’s Republic of China

**Keywords:** Dual-dynamic-bond cross-linked, Multifunctional hydrogel, Oxidative stress-related disease, Minimally invasive therapy

## Abstract

Developing smart hydrogels with integrated and suitable properties to treat intervertebral disc degeneration (IVDD) by minimally invasive injection is of high desire in clinical application and still an ongoing challenge. In this work, an extraordinary injectable hydrogel PBNPs@OBG (Prussian blue nanoparticles@oxidized hyaluronic acid/borax/gelatin) with promising antibacterial, antioxidation, rapid gelation, and self-healing characteristics was designed via dual-dynamic-bond cross-linking among the oxidized hyaluronic acid (OHA), borax, and gelatin. The mechanical performance of the hydrogel was studied by dynamic mechanical analysis. Meanwhile, the swelling ratio and degradation level of the hydrogel was explored. Benefiting from its remarkable mechanical properties, sufficient tissue adhesiveness, and ideal shape-adaptability, the injectable PBNPs containing hydrogel was explored for IVDD therapy. Astoundingly, the as-fabricated hydrogel was able to alleviate H_2_O_2_-induced excessive ROS against oxidative stress trauma of nucleus pulposus, which was further revealed by theoretical calculations. Rat IVDD model was next established to estimate therapeutic effect of this PBNPs@OBG hydrogel for IVDD treatment in vivo. On the whole, combination of the smart multifunctional hydrogel and nanotechnology-mediated antioxidant therapy can serve as a fire-new general type of therapeutic strategy for IVDD and other oxidative stress-related diseases.

**Supplementary Information:**

The online version contains supplementary material available at 10.1186/s12951-022-01633-0.

## Introduction

Low back pain, a chronic disease, increases with age and causes disability and high medical costs around the world [1], whose mainspring is intervertebral disc degeneration (IVDD) [2]. It is known that the perplexing structure of IVD is mainly composed of annulus fibrosus (AF), nucleus pulposus (NP), and cartilage endplate [3], and the AF derives from the mesenchyme, while the NP comes from the notochord. The basic component of AF is collagen fiber, which is of excellent toughness; however, NP is gelatinous, isotropic, and rich in water. Compared with other human tissues, the disc cells are prone to degenerative insomuch as low oxygen, pH, glucose, and high load fluctuation [4]. Unfortunately, disc cells cannot repair themselves on account of limited regenerative competency. In the early stage of IVDD, the enclosed environment makes the external stem cells difficult to exert potential regenerative ability on the NP. In the later stage as the degeneration becomes severe, the AF tear happens, and neovascularization and neoinnervation will occur in the NP. Inflammation caused by the newly-colonized immune cells further harm the microenvironment and accelerates tissue degeneration. Thus, deteriorating microenvironment and low self-regenerative ability of the IVD are primary obstacles for regeneration [5]. So, spinal fusion becomes the conventional therapeutic manner for patients that had failures in previous conservative treatment. Nevertheless, this operation could lead to degenerative changes at adjacent segments [6]. To date, discectomy has emerged as an alternative procedure to spinal fusion. But, the NP of disc could continue to herniate because surgery cannot supply the lost NP [7].

In view of heavy economic burden and finiteness of surgical intervention, developing new therapeutics for the treatment of IVDD is a substantial need. Stem cells implantation can relieve IVDD or regenerate degenerated disc [8, 9]; however, transplanting cells into the disc encounters challenges such as extremely low survival rate, complicated preparation, and unknown long-term results. Gene therapy might be an alternative strategy for amelioration of IVDD, but it is still limited by low gene transfection efficiency [10]. On the other hand, a variety of sicknesses including glomerulonephritis [11], myocardial infarction [12], sepsis [13], and inflammatory bowel disease (IBD) [14], are closely related with superfluous reactive oxygen species (ROS) generation [15, 16]. Similarly, excessive oxidative stress exacerbates the progression of IVDD by including inflammatory cytokine naissance, boosting senescence and apoptosis of NP cells, invoking MAPK/NF-κB signaling pathways, and aggravating mitochondrial dysfunction [17–19]. So, there lies great demand to develop new eutherapeutic approaches and great significance to explore effective methods to treat IVDD based on the oxidative stress microenvironment.

**Scheme 1.**
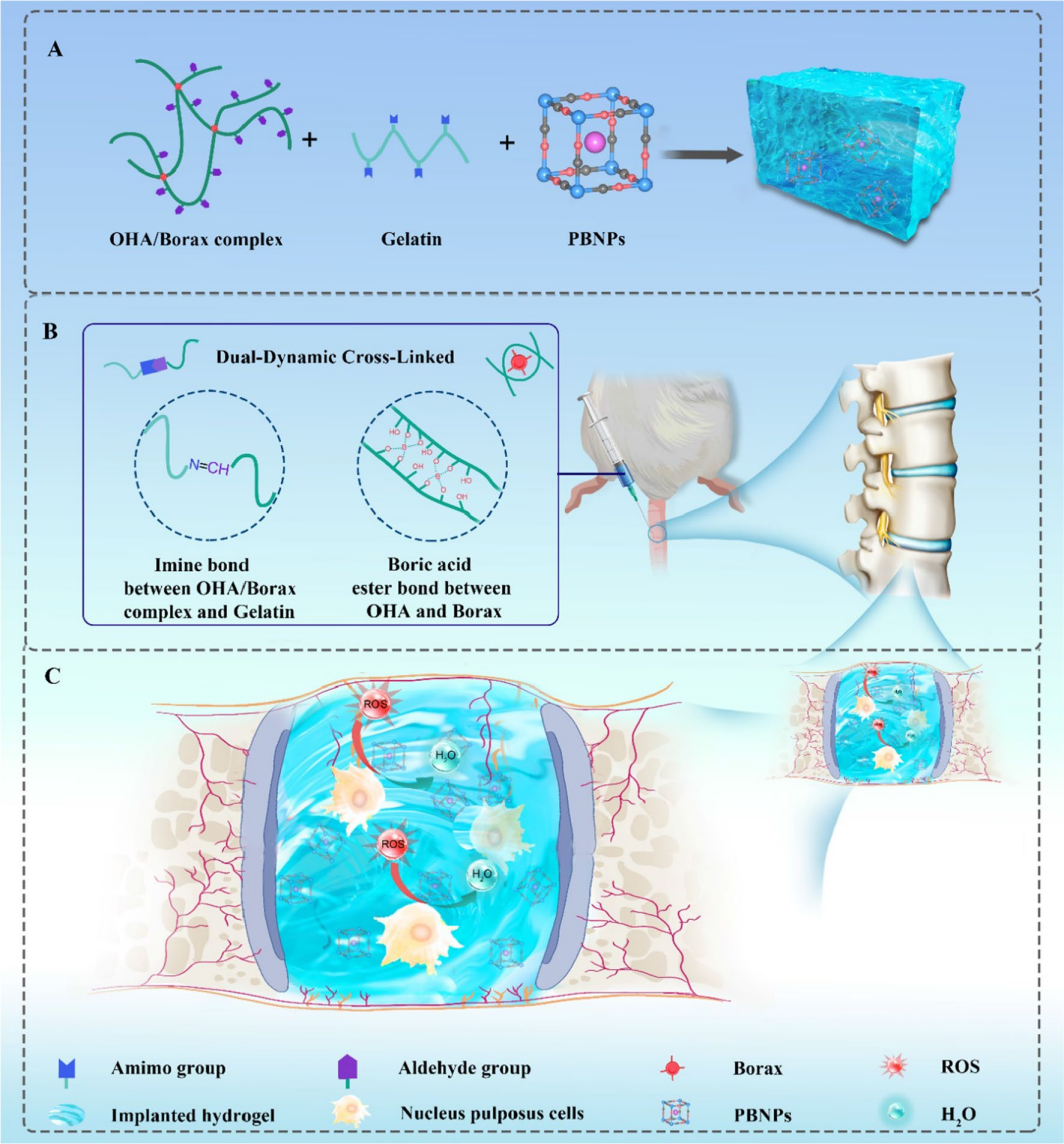
Concept illustration. **A** Fabrication scheme of the PBNPs@OBG hydrogel. **B** Schematic of hydrogel structure mainly composed by imine bond and boric acid ester bond. **C** Mitigation of oxidative stress microenvironment within NP cells and notable amelioration of IVDD caused by PBNPs-dotted hydrogel

Currently, antioxidant supplementation such as epigallocatechin gallate, resveratrol, and ferulic acid have shown certain curative effects in the treatment of IVDD [20–22]. Prussian blue nanoparticles (PBNPs), which had been approved by the Food and Drug Administration (FDA) as antidote for cesium and thallium toxicity, possessing biocompatibility, photothermal properties, and antioxidant abilities [23, 24]. have been extensively applied in biomedical fields including biosensors [25], cancer theranostic [26], tumor therapy [27], and antidote for poisoning [28]. As a promising candidate for treatment of ROS-related disease such as stroke [29], osteoarthritis [30], and acute pancreatitis [31]. The application of PBNPs in IVDD repair still remains unexplored, and how PBNPs can eliminate endogenous ROS (H_2_O_2_) has not been clarified. But, systemic application of PBNPs is improper for IVDD due to the avascular structure of disc [32]. Although liquid injection in situ might be a much more appropriate way for disc degeneration, the lumbar intervertebral disc is located between the vertebral bodies, bearing much more pressure than the thoracic and lumbar vertebrae, so appropriate mechanical properties are required of materials for intervertebral disc tissue engineering usage [33]. Surprisingly, the introduction of injectable adhesive hydrogel not only copes with the puzzle that liquid therapeutics are inclined to leak out from the disc and subsequently lead to a series of complications [34], but also provides a certain degree of load-bearing capacity for the intervertebral disc and contributes to restore natural structure, biological function, and mechanical properties of disc [35]. Hydrogel encapsulated with PBNPs also enables sustainable release and long-term retention of loaded substances, and the enhanced retention of hydrogel can be achieved due to the adhesiveness through proper materials design [36], which provides a great prospect for substitution of degenerated intervertebral disc with a tissue-engineered plasticated disc. More importantly, injecting hydrogel into the degenerated intervertebral disc belongs to a minimally invasive surgical procedure, which also has the risk of intervertebral disc infection. Once an infection occurs in the disc, a series of complications such as limited motion, immobile and point tenderness may came up [37]. Particularly, it is vital to tackle the infection matter during the process of minimally-invasive injection at the meantime [38]. What’s more, new synthetic biomaterials with the ability of self-healing mechanical is essential for improving performance and prolonging the life of implants [39, 40]. To summarize, we hypothesize that multifunction integrated hydrogel loaded with PBNPs can efficiently remove the ROS in the oxidative stress microenvironment of IVDD via sustainedly releasing PBNPs and achieve significant attenuation of IVDD (Scheme 1).

Herein, we developed an advanced multifunctional injectable hydrogel for treatment against IVDD. This multifunctional dual cross-linked hydrogel was fabricated by integrating two functional modules including OHA/borax-gelatin (OBG) and PBNPs. PBNPs served as efficient ROS scavengers. Borax and oxidized hyaluronic acid (OHA) were utilized to form borate-diol complexation [41, 42], and further reacted with gelatin via Schiff’s base reaction. The dynamic imine bond and boric acid ester bond endowed the hydrogel with excellent self-healing and injectable properties [43–46]. As expected, the hydrogel exhibited pyrotechnic antibacterial effect, antioxidant capacity, biocompatibility, tissue adhesiveness, and suitable mechanical properties. Compared with other HA based hydrogels with antioxidant activities which were applied in would healing, our hydrogels have the characteristics of injectable, antibacterial and self-healing, and are more suitable for application in intervertebral disc degeneration environment [47]. To the best of our knowledge, the PBNPs-loaded hydrogel with these multi-functionalities for IVDD treatment has not been reported. The hydrogel with overall network structure will show certain viscosity or elasticity, which is similar to the nature of NP of intervertebral disc, making it a splendiferous candidate for tissue-engineered NP utilization.

## Materials and methods

### Materials

Sodium hyaluronate was purchased from Yuanye Bio-Technology Co. Ltd (Shanghai, China). Ethylene glycol, sodium periodate (100–150 kDa), rhodamine B, and polyvinyl pyrrolidone were purchased from Aladdin (Shanghai, China). 2, 7-dichlorodihydrofluorescein diacetate (DCFH-DA), 4′, 6-diamidino-2-phenylindole (DAPI), potassium ferricyanide, and gelatin were obtained from Sigma. Cy5 was bought from Ruixibio (Xi’an, China). Cell Count Kit-8 (CCK-8) and Live/dead kit were acquired from Dojindo Molecular Technologies (Kumamoto, Japan). The JC-1 dye and Annexin V-FITC Apoptosis Detection Kit was bought from Solarbio (Solarbio, China). Antibodies of MMP3 (ab52915) and were purchased from Abcam (Abcam, UK). Collagen II (sc-52658) was purchased from Santa cruz (Santa cruz, USA). Aggrecan (13,880-1-AP), MMP13 (18,165-1-AP) and SOX9 (67,439-1-Ig) was purchased from Proteintech (Proteintech, China).

### Preparation of PBNPs

Polyvinyl pyrrolidone (4 g), potassium ferricyanide (396 mg), and HCl (100 mL, 1 M) were mingled under magnetic stirring until a limpid solution was acquired, and then the liquor was put into an oven at 80 °C for 24 h. The color of the solution changed from yellow to deep blue. Subsequently, by centrifuging at 25,000 rpm for 15 min and washing with distilled water and acetone for several times, PBNPs were obtained [48].

### Characterizations

The morphology of PBNPs was observed by Transmission electron microscopy (TEM, Hitachi HT-7700, Japan) and Scanning electron microscopy (SEM, SU-8010, Hitachi, Japan). The crystal properties were evaluated by X-ray diffraction (XRD, Bruker D8 advance Diffracto meter, Bruker, Germany). The chemical status and chemical bonds were tested by X-ray photoelectron spectroscopy (XPS, ESCALAB 250Xi, Thermo Scientific, USA). The chemical structure of the HA and OHA was verified by Fourier transform infrared (FT-IR, Vertex 70, Bruker Optics, Ettlingen, Germany), and ^1^H-NMR spectroscopy (Bruker AVANCE III 400 MHz, Switzerland), samples were dissolved in D_2_O and transferred to 400 MHz NMR glass tubes spectra acquisition. UV–vis-NIR spectra were recorded on UV-3600 (Shimadzu, Japan). The size of PBNPs was assessed using dynamic light scattering (DLS) on Zetasizer Nano ZS90 (Malvern instruments Ltd, Malvern, UK).

### Preparation of OHA/borax-gelatin hydrogel containing PBNPs

HA (2 g) was dissolved in distilled water (200 mL) under magnetic stirring. Sodium periodate (NaIO_4_) solution was added while stirring, and the molar ratio of NaIO_4_ to HA was 1: 1 (the molar ratio of NaIO_4_ to repeating sugar unit of HA). After stirring for 24 h, the reaction was suspended by adding ethylene glycol (2 mL) into the solution. The obtained solution was dialyzed in dialysis bags (MWCO 8000–12,000 Da) for 5 days and lyophilized. Hydrogels were prepared using a 25% solution of gelatin and 10% solution of OHA in 0.1 M borax [49]. For construction of the PBNPs@OBG, PBNPs were suspended in OHA and gelatin solutions before the gelation process. The gelation time was about 5 s and the hydrogel was stood for 12 h to form the final product. The PBNPs content in each hydrogel (200 μL) was 0.2 μg.

### Establishment of rat IVDD model

All animal experiments were approved by the Animal Ethics Committee, Sir Run Run Shaw Hospital, Zhejiang University. Sprague Dawley rats of 10 weeks age were anesthetized by pentobarbital (2%, *W/V*, 40 mg kg^−1^). After rat tails were disinfected, 21 G needle was vertically punctured into C6-7 IVDs to induce degeneration. To guarantee the trauma was caused, the depth of puncture was controlled at 5 mm. The needle was rotated 360° and maintained in the position for 30 s. Then, the rats were randomly divided into 5 groups and administrated with corresponding treatments: Control group with needle puncture; Acupuncture group with needle puncture and injection of PBS; PBNPs@OBG group with needle puncture and PBNPs@OBG injection; PBNPs group with needle puncture and PBNPs injection; OBG group with needle puncture and OBG injection. For all the situations, 20 μL of the materials were injected into the discs using a 1 mL injector (26 G). Rats were placed in a warm place and monitored daily after surgical procedure. After 4 and 8 weeks, all the rats were euthanized with an overdose of chloral hydrate.

### Biosafety evaluation in vivo

For histopathological analyses, rats were sacrificed after 4 and 8 weeks post-injection to collect the major organs (heart, lung, spleen, liver, and kidney), the tissues were fixed in 4% paraformaldehyde and stained with hematoxylin and eosin. Meanwhile, 600 μL of the blood from the rats were collected for blood routine examinations and standard blood biochemical. Blood routine examinations parameters, including white blood cells, red blood cells, and platelets.

### Histological and immunofluorescence analyses

The IVDs of each rat were taken out and fixed in formalin for 2 days. Then IVDs decalcified in EDTA for 30 days. As-prepared samples were cut into 3 μm sections, slices were baked in thermostat at 60 °C overnight, then stained with H&E, Safranin-O/Fast Green, and Alcian Blue. For Alcian Blue staining, samples were treated with Alcian acidizing buffer for 6 min, then stained with Alcian staining buffer for 20 min. H&E, Safranin/O-Fast Green were processed in accordance with standard laboratory protocols. Two observers estimated the cellularity and morphology in a blinded fashion. For Immunofluorescence assessment, after permeabilizing with Triton X-100 (0.5%) and blocking with 10% goat serum, the samples were incubated with the primary antibody (1: 200) (COL II, MMP3, MMP13, Aggrecan) overnight at 4 °C. After washing with PBS for 3 times. Samples were stained with the secondary antibody Alexa Fluor 488 (1: 200) for 1 h at room temperature, the nucleus were stained with 4’,6-diamidino-2-phenylindole (DAPI), then observed by a confocal microscope.

### Statistical analysis

All the data were shown as mean ± standard deviation. Statistical differences were analyzed using one-way analysis of variance or two-tailed nonpaired Student’s t-test (*P < 0.05, **P < 0.01, ***P < 0.001).

## Results and discussion

### Preparation and characterization of PBNPs

**Fig. 1:**
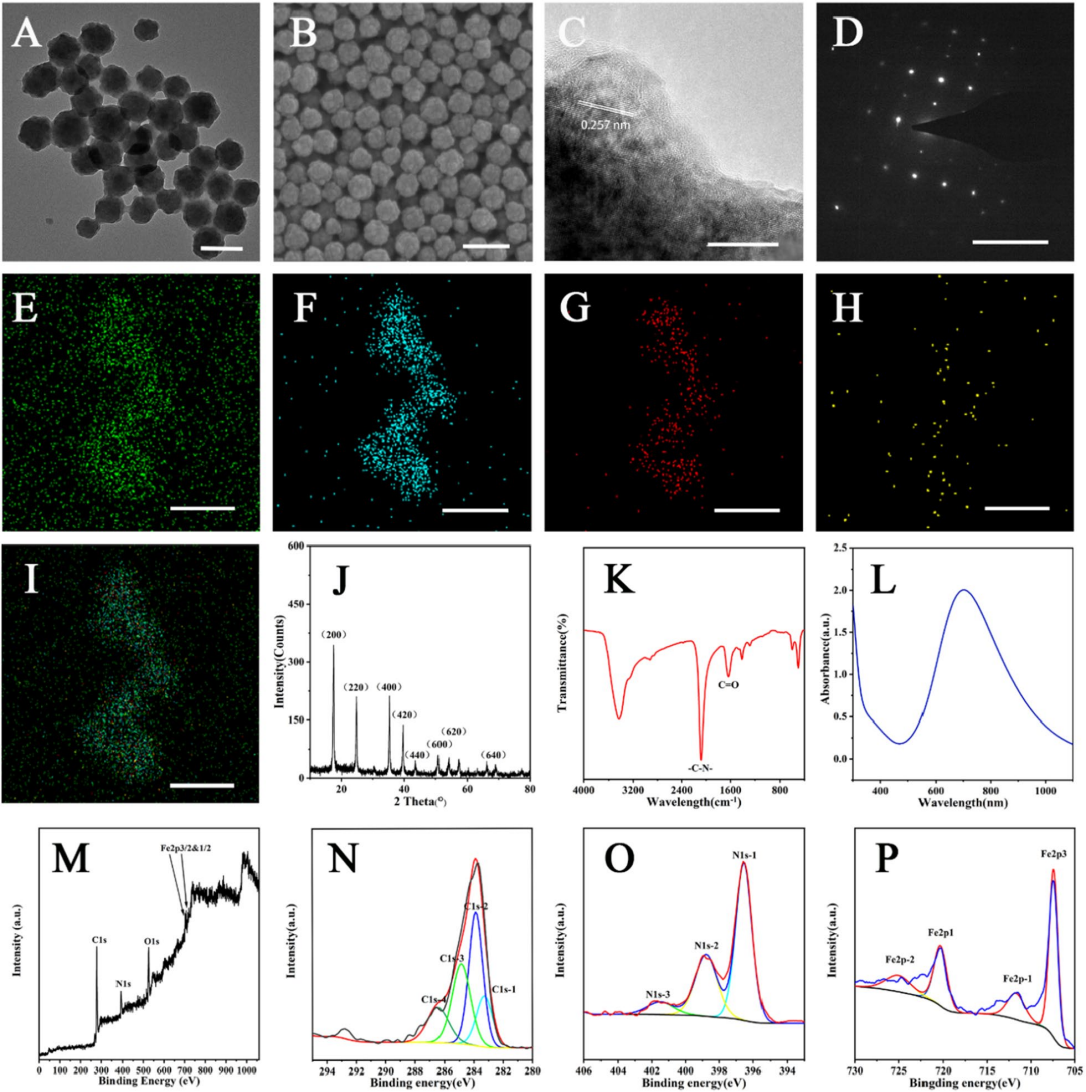
Morphology and characterization of PBNPs. **A** TEM image of PBNPs. Scale bar = 100 nm. **B** SEM micrograph of PBNPs. Scale bar = 200 nm. **C** High resolution transmission electron microscopy (HRTEM) image. Scale bar = 10 nm. **D** SAED of PBNPs. Scale bar = 10 nm. **E** − **I** Element mapping of PBNPs. Green: C, Blue: Fe, Red: N, Yellow: K, I: Merged. Scale bar = 200 nm. **J** XRD pattern of PBNPs. **K** FTIR spectroscopy. **L** UV–vis-NIR spectrum. **M** Survey XPS spectra, and **N** − **P** Element XPS spectra (carbon, nitrogen, and iron) of PBNPs

PBNPs were prepared by hydrothermal method. Potassium ferricyanide and polyvinylpyrrolidone were dissolved in hydrochloric acid with a magnetic agitator. After reacted overnight in an 80 °C oven, PBNPs were obtained by centrifugation and washed. In the reaction, polyvinylpyrrolidone served not only as a reducing agent, but also as a stabilizer (Additional file 1: Figure S1). Transmission electron microscopic (TEM) demonstrated that as-synthesized PBNPs with spherical structure could be well dispersed in deionized water and the average diameter was ~ 76 nm (Fig. 1A), whose hydrodynamic size was measured to be around 120 nm (Additional file 1: Figure S2). The observation result of scanning electron microscope (SEM) was well consisted with TEM results (Fig. 1B). Electron diffraction pattern of the selected region also revealed that PBNPs had discontinuous lattice stripes, and the lattice fringe spacings were 0.45, 0.33, and 0.25 nm, respectively (Fig. 1C), which was well coincident with the results of characteristic peak diffraction surface indicated by X-ray diffraction (XRD). XRD results confirmed that structural formula of the PBNPs was Fe_3_[Fe(CN)_6_]_2_·(JCPDS No. 73–0687, Fig. 1J) [30]. Selected area electron diffraction (SAED) pattern indicated good crystallinity of the PBNPs (Fig. 1D), and main compositions of the PBNPs were revealed to be Fe, C, N, and K based on element mapping results (Fig. 1E–I). And Fourier transform infrared spectroscopy (FT-IR) spectrum data demonstrated the PBNPs had a characteristic absorption peak at 2086 cm^−1^, which proved that Fe^II^-CN-Fe^III^ existed in the structure of PBNPs (Fig. 1K). PBNPs exhibited characteristic absorption peak at a wavelength of ~ 706 nm, which can be attributed to the charge transfer band from Fe^II^ to Fe^III^ (Fig. 1L). X-ray photoelectron spectroscopy (XPS) survey spectra showed that the elemental composition of C1s, N1s, O1s Fe2p3/2 and 1/2 (Fig. 1M). The peaks of C1s-1 with binding energy are 284.33 eV, N1s-1 with binding energy of 396.98 eV, and the peaks of Fe element suggested the chemical group of formation of Fe-CN-Fe structures (Fig. 1N–P). Differential scanning calorimetry and thermogravimetry analyses were performed at a heating rate of 10 °C min^−1^ in air atmosphere ranging from 37 to 1000 °C using a thermal analyzer. The PBNPs showed a 11.77% mass loss between 37 and 135 °C, which should be attributed to the elimination of water from PBNPs, while the second weight loss part in the temperature range of 135 − 1000 °C was due to the decomposition of the polyvinylpyrrolidone and transformation of Prussian blue into ferric oxide, the remaining mass of ferric oxide is 41.93%, which was the end product of decomposition (Additional file 1: Figure S3). The adsorption curve of PBNPs exhibits hysteresis looped when relative pressure ranges from 0.0 to 1.0 (Additional file 1: Figure S4).

### Preparations and characterizations of the OBG and PBNPs@OBG Hydrogel

**Fig. 2:**
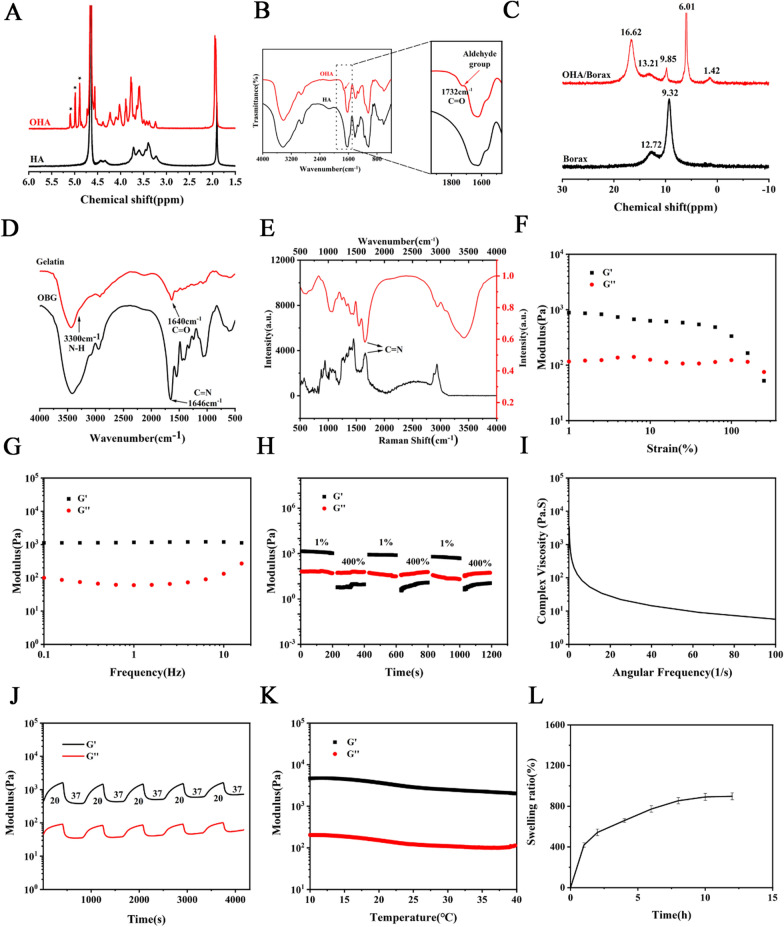
**A** 1H-NMR spectra of HA and OHA, showing the existence of aldehyde groups in modified HA. **B** FT-IR spectrum of HA and OHA. **C** 11B NMR spectra of borax and OHA/borax complex, revealing the formation of borate–diol complexation. **D** FT-IR spectra of gelatin and OBG for demonstrating the hydrogel formation. **E** Raman (black) and FT-IR (red) spectrometry analysis of OBG hydrogel. **F** Oscillatory strain − sweep test of the PBNPs@OBG hydrogel at 37 °C (ω = 10 rad s^ − 1^). **G** Frequency dependent rheology of the PBNPs@OBG hydrogel at 37 °C (ε = 1%). **H** Step − strain test of the PBNPs@OBG hydrogel at low (1%) and high (400%) strains to illustrate the self-healing properties at 37 °C (ω = 10 rad s^ − 1^). **I** Shear-thinning behavior of the PBNPs@OBG hydrogel indicated by steady-shear rheology. **J** Oscillatory time sweep of the PBNPs@OBG hydrogel between 37 and 20 °C. **K** Oscillatory temperature sweep of the PBNPs@OBG hydrogel. **L** Swelling ratios of the PBNPs@OBG hydrogel varied with time (n = 3)

OHA was synthesized by oxidizing hydroxyl groups of hyaluronic acid to aldehyde groups by sodium periodate, which was confirmed by ^1^H NMR and FT-IR. The ^1^H NMR of HA and OHA was similar, while the characteristic peak corresponding to aldehyde group appeared at 4.8–5 ppm (denoted as stars) [50, 51], which was absent in the unmodified HA (Fig. 2A). A newly appeared peak at 1732 cm^−1^ in the OHA spectrum was associated with the C = O stretch (Fig. 2B) [50]. The amine group in gelatin can form imine bond with the aldehyde in OHA in the presence of borax. Adding borax to OHA formed the borate-diol complexation, resulting in the connection of HA chains at multiple sites, thus accelerating the gelation process. The ^11^B NMR spectra of borax and OHA-complexed borax can be seen in Fig. 2C. The standard sample of borax had two peaks, which were 12.72 and 9.32 ppm. The peak located at 12.72 ppm was B_3_O_3_(OH)_3_^−^ and the peak at 9.32 ppm represented the equilibrium of B(OH)_3_ and B(OH)_4_^−^. The position of 1.42 ppm can be the six membered single chelate product with OHA. Peak at 16.62 ppm was the unreacted B(OH)_3_. The chemical shift at 13.21 ppm may be attributed to B_3_O_3_(OH)_4_^−^, and 6.01 and 9.85 ppm should be the five membered single chelate product and double chelate product of B(OH)_4_^−^ reacted with OHA, where a similar observation was covered previously [52]. The FT-IR results of OHA/borax-gelatin (OBG) and gelatin were displayed in Fig. 2D. Peaks at 3300 and 1640 cm^−1^ represented N–H and C = O stretch, respectively. The peak around 1646 cm^−1^ (C = N) appeared in OBG hydrogel indicated the success of Schiff’s base reaction [53], which was also confirmed by Raman spectrometry analysis (Fig. 2E) [54, 55]. The mixture of prepared OHA/Borax complex and gelatin aqueous solution presented in the sol state at room temperature. The obtained PBNPs were then added to the premix of OHA/Borax complex or gelatin along with consistent stirring. Surprisingly, the solution experienced sol − gel transition at 37 °C as one aqueous solution was added into the other (Additional file 1: Figure S5). As displayed in Additional file 2: Video S1) the gelation time was within 5 s, and such ultrafast gelation facilitated its direct use in the injectable application. Both of the OBG and PBNPs@OBG samples presented three-dimensional highly-porous inner structures (Additional file 1: Figure S6). Elements of the PBNPs@OBG including C, N, O, B, and Fe, were all verified in the energy dispersive spectrometer mapping images. Fe as characteristic element of the PBNPs, its uniform dispersion revealed a homogeneous encapsulation of PBNPs within the OBG hydrogel (Additional file 1: Figure S7). Rheological tests have been widely accepted nowadays to evaluate mechanical properties of the hydrogels, such as self-healing ability, shear thinning behavior, and thermoresponsive reversibility, and so forth [56]. Firstly, we performed strain amplitude sweep test. The strain sweep of the hydrogel on a rheometer (37 °C, *ω* = 10 rad s^−1^) witnessed the curves of storage modulus and loss modulus intersect at about 220% strain, which meant the critical point (Fig. 2F). Within the overall rotational frequency scale, G’ was consistently higher than G’’, indicating a solid state of the PBNPs@OBG hydrogel. It became liquid-like along with additional increase in strain of above 220%, which implied network ruptures at high strain (Fig. 2G). The step − strain test was performed to evaluate an autonomous healing performance of the dynamic PBNPs@OBG hydrogel at 37 °C, which referred to the ability of maintaining hydrogel structure completeness after suffering an action of external force. When applying a high strain of 400%, a fluid-like behavior was observed, where the hydrogels were converted into a sol state and *G’*< *G’’* was manifested on the graph. While a low strain of 1% was conducted, *G’* and *G’’* almost restored to their original values even after three cycles, confirming that this recovery profile was repeatable (Fig. 2H). Additional, as the shear rate increased, the complex viscosity reduced, declaring a shear-thinning behavior of the PBNPs@OBG hydrogel (Fig. 2I). Dynamic temperature sweep was carried out as well, when the temperature varied between 37 and 20 °C, the *G’* and *G’’* value recovered quickly and reversibly, purporting the thermoresponsive reversibility of the hydrogels (Fig. 2J). The *G’* and *G’’* dropped slightly while the value of *G’* was still higher than that of *G’’* as the temperature rising, indicating an existence of solid state (Fig. 2K). The swelling rate of hydrogel was detected in PBS, and the PBNPs@OBG hydrogel reached swelling equilibria (892% ± 159%) at about 10 h after immersing in PBS (Fig. 2L). Degradation test was performed in PBS (pH = 7.4 and pH = 6.5) at 37 °C, the whole degradation process would continue for about 30 days in pH 7.4 PBS. Both boric acid ester bond and Schiff base bond are acid-sensitive [57, 58]. It is reported that pH 6.5 medium was used to simulate acidic stress as a result of the local overactive inflammatory response in the IVD environment [59]. The hydrogel could be fully degraded within about 24 days in pH 6.5 PBS, indicating the pH-responsive degradation property in vitro (Additional file 1: Figure S8).

### Injectable, reformable, self-healing, and mechanical properties of the PBNPs@OBG hydrogels

**Fig. 3:**
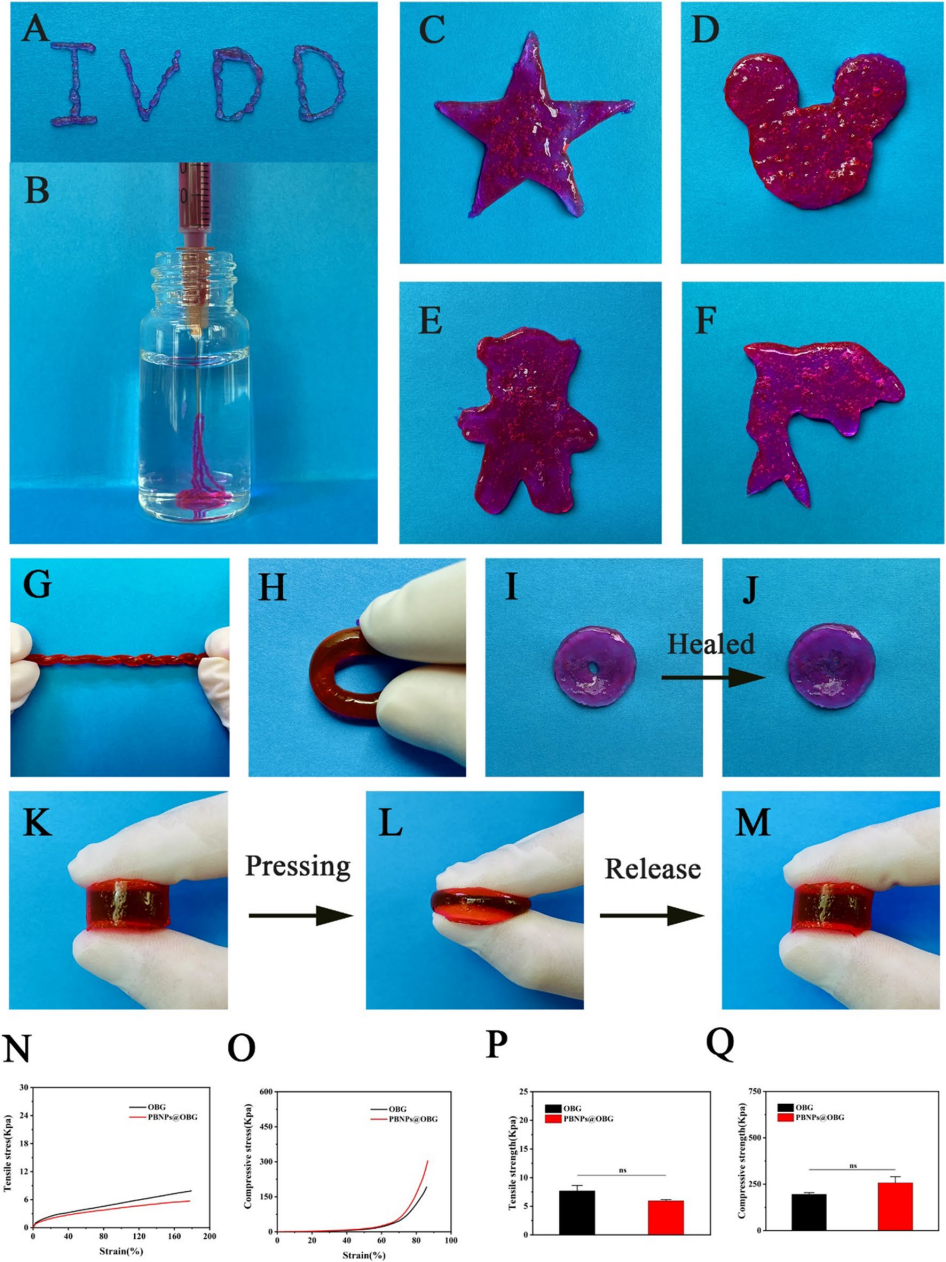
**A**, **B** Injecting process of the PBNPs@OBG to demonstrate shear-thinning injectability and good writing ability. **C** − **F** Photographs of the hydrogel's phenotypic plasticity, including star, Mickey Mouse, little bear, and dolphin. **G** Twisting shape and **H** bending shape of the PBNPs@OBG. **I**, **J** The process of self-healing. **K** − **M** Original, pressing, and recovery shapes of the PBNPs@OBG, indicating peachy flexibility of the hydrogel by bearing deformation under repeated stretching and compression conditions without breaking. **N** Representative tensile and **O** compression stress − strain curves of the OBG and PBNPs@OBG. **P** Tensile and **Q** compressive strengths of the OBG and PBNPs@OBG (n = 3, *ns* not significant)

The rhodamine B-dyed dual-dynamic-bond cross-linked hydrogel was injectable, which could be administered by a syringe (Fig. 3A–B). The superb shape-adaptability brings the hydrogel appropriate for diseased disc with irregular shapes. The hydrogel was nimble to be remodeled into any intricate appearance discretionarily, such as dolphin, bear, star, and Mickey Mouse (Fig. 3C–F). The PBNPs@OBG hydrogel was able to tolerate twisting and bending, indicating flexible properties (Fig. 3G–H). A hole was created in the center of the hydrogel, and the hydrogel recovered from the breakage after half an hour (Fig. 3I–J), demonstrating the self-healing performance of the hydrogel. Simultaneously, cylindrical shape of the hydrogel restored to its original state within 1 s after pressing (Fig. 3K–M), indicating peachy flexibility. We investigated the compression and tensile properties of the implanted hydrogels by a dynamic mechanical analyzer at 37 °C. Taken together, the OBG and PBNPs@OBG hydrogels both reached up to 1.8 times of the initial length and could be compressed to ten percent of initial thickness without rupture. The tensile strengths of the OBG and PBNPs@OBG hydrogel were 7.69 ± 0.93 and 5.93 ± 0.22 kPa and the compressive strength achieved 195.33 ± 9.29 and 257.33 ± 33.00 kPa, respectively (Fig. 3N–O and Additional file 1: Figure S9). There was no significant difference between the OBG and PBNPs@OBG in the above tests (Fig. 3P–Q).

### Adhesion, antibacterial ability of hydrogels and long term retention of PBNPs in the PBNPs@OBG hydrogel

**Fig. 4:**
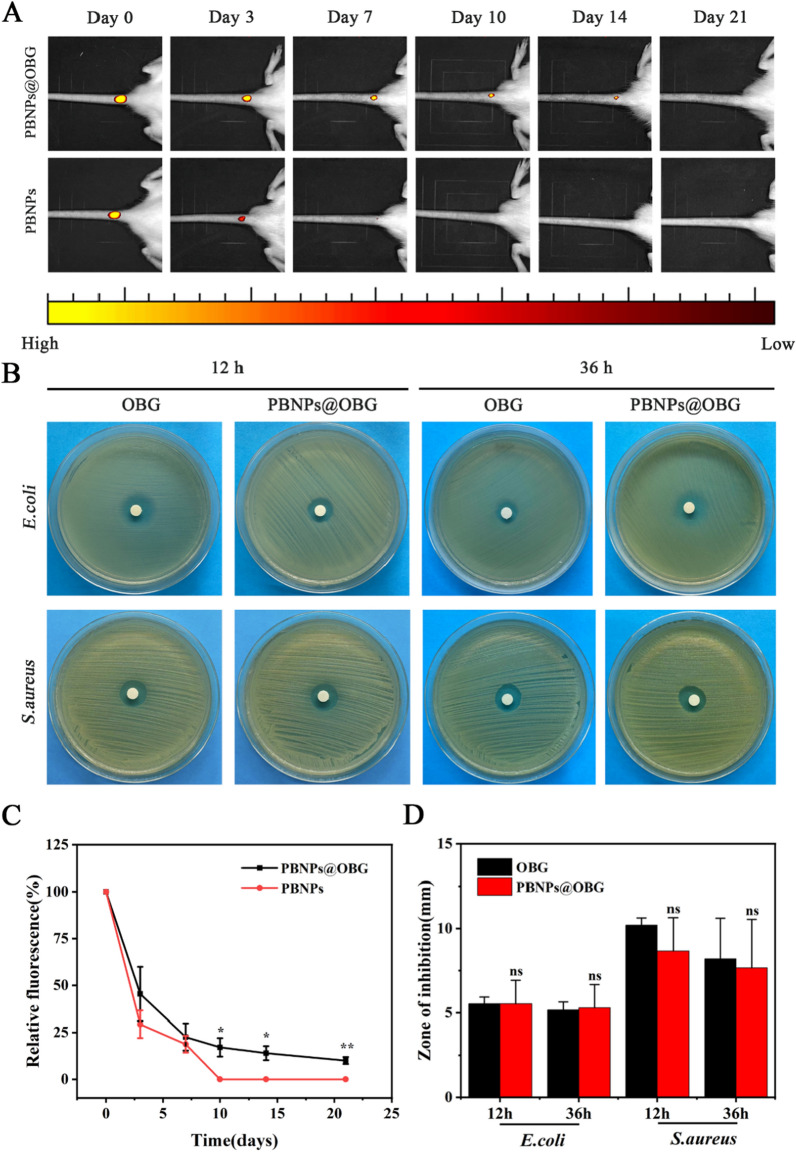
**A** Representative fluorescence image after Cy5-labeled free PBNPs solution and Cy5-labeled PBNPs@OBG hydrogel were injected into disc sites using IVIS over time. **B** Inhibition zones of the OBG and PBNPs@OBG on S. aureus and E. coli after 12 and 36 h using agar diffusion test. **C** Quantitative analyses of free PBNPs and PBNPs@OBG group tests via IVIS. **D** Inhibition zone diameters for S. aureus and E. coli in the OBG and PBNPs@OBG groups (n = 3, *P < 0.05, **P < 0.01, ns: not significant)

PBNPs were labeled with Cy5 to validate the efficient retention and sustained release in vivo, Cy5-labeled PBNPs and Cy5-labeled PBNPs@OBG were injected into rat discs. PBNPs group retained faint fluorescence after 7 days; whereas, fluorescence signal of the PBNPs@OBG group was lasted for a long period time of 21 days and then was disappeared. OBG@Cy5-labeled PBNPs maintained stable and sustainable release owing to the adhesive behavior of hydrogel (Fig. 4A, C). As shown in Additional file 1: Figure S10), two pieces of glass were adhered together by the PBNPs@OBG hydrogel, and it could endure at least 500 g weight. Next, tissue adhesion was divided into 5 groups and investigated via lap-shear adhesion tests, both the OBG and PBNPs@OBG showed exceptional adhesiveness, which refrained divulgation of the hydrogel and contributed to everlasting retention of PBNPs in the PBNPs@OBG hydrogel. Borax, an FDA approved material, has been covered to be an antibacterial agent [60]. In this research, we detected anti-*S. aureus* and anti-*E. coli* activities by agar diffusion method. As we anticipated, both the OBG and PBNPs@OBG had decent antibacterial activities against gram-positive *S. aureus* and gram-negative *E. coli* thanks to the presence of borax. The diameter of bacterial inhibition halos around the OBG and PBNPs@OBG were 10.2 ± 1.4 and 8.7 ± 2.0 mm for *S. aureus*. The zone diameter in the OBG and PBNPs@OBG group reached 5.5 ± 0.4 and 5.5 ± 1.4 mm for *E. coli* after 12 h. The results of 36 h were similar to those of 12 h (Fig. 4B, D).

### Antioxidant efficiencies

**Fig. 5:**
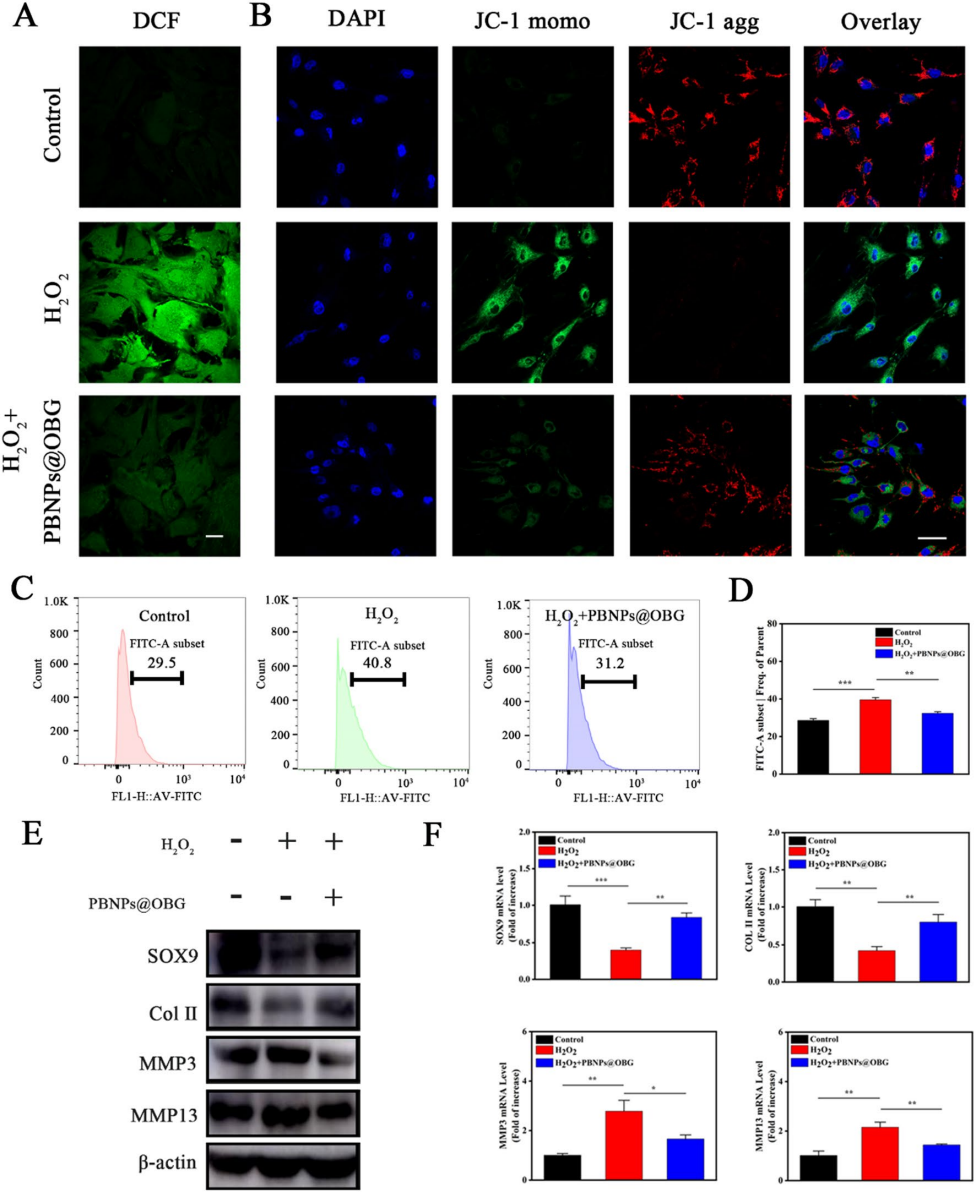
**A** Fluorescence images showing reduction of intracellular ROS with ROS staining by DCFH-DA probe. **B** Effect of the PBNPs@OBG on mitochondrial membrane potential in NP cells by JC-1 staining. Scale bar = 50 µm. **C**–**D** Effect of the PBNPs@OBG on ROS levels after treated with H2O2 determined by flow cytometry on NPs. **E**–**F** Expressions of SOX9, collagen II, MMP3, and MMP13 were determined by WB **E** and qRT-PCR **F** of NPs in vitro (n = 3, *P < 0.05, **P < 0.01, ***P < 0.001)

The production of excessive ROS in NP cells was the main cause of degeneration during the progress of IVDD. We investigated whether PBNPs could react with the endogenous ROS (H_2_O_2_) in vitro firstly. When PBNPs was mixed with H_2_O_2_, bubbles appeared (Additional file 1: Figure S11), which meant generation of O_2_. Then we introduced the PBNPs into our OBG hydrogel to observe the scavenging effect in vivo. The dichlorodihydro-fluorescein diacetate (DCFH-DA) was used as a probe to test the ROS generation. H_2_O_2_ has been extensively employed to induce an excessive ROS environment in intervertebral disc degeneration [15]. Apparent fluorescence quenching was observed when treated with the PBNPs@OBG (Fig. 5A). Flow cytometry results also demonstrated an apparent ROS elimination effect (Fig. 5C, D). JC-1 was chosen as a mitochondrion specific lipophilic cationic fluorescence dye to detect the effect of the PBNPs@OBG on mitochondrial dysfunction in H_2_O_2_-induced NP cells [61]. The mitochondrial function was characterized by mitochondrial membrane potential (MMP). The mitochondrial was destroyed in H_2_O_2_-induced group, which manifested decreased MMP. JC-1 exists in the form of green fluorescent monomer chiefly. The other way around, cells treated with the PBNPs@OBG produced higher red fluorescence and lower green fluorescence (JC-1 monomers), indicating the restoration of MMP (Fig. 5B and Additional file 1: Figure S12). Furthermore, flow cytometry disclosed that the apoptosis of NPs induced by H_2_O_2_ was effectively mitigated by the PBNPs@OBG (Additional file 1: Figure S13). Overexpression of matrix metalloproteinases (MMPs) such as MMP3 and MMP13 can lead to degradation of collagen II, which is the major components of ECM. According to Western blot (WB) and quantitative real-time polymerase chain reaction (qRT-PCR) results, after H_2_O_2_ stimulation, the expressions of MMP3 and MMP13 increased compared to the Control group, the expression of collagen II and SOX9 were down-regulated. However, the PBNPs@OBG delayed the increased expression of MMPs and reduced degradation of ECM, and thus retarding the progression of IVDD (Fig. 5E, F). These results lead to the conclusion that the PBNPs@OBG could ameliorate H_2_O_2_-induced excessive ROS in cultured NP cells.

### Density functional theory calculations and in vivo experiments using an IVDD rat model

**Fig. 6:**
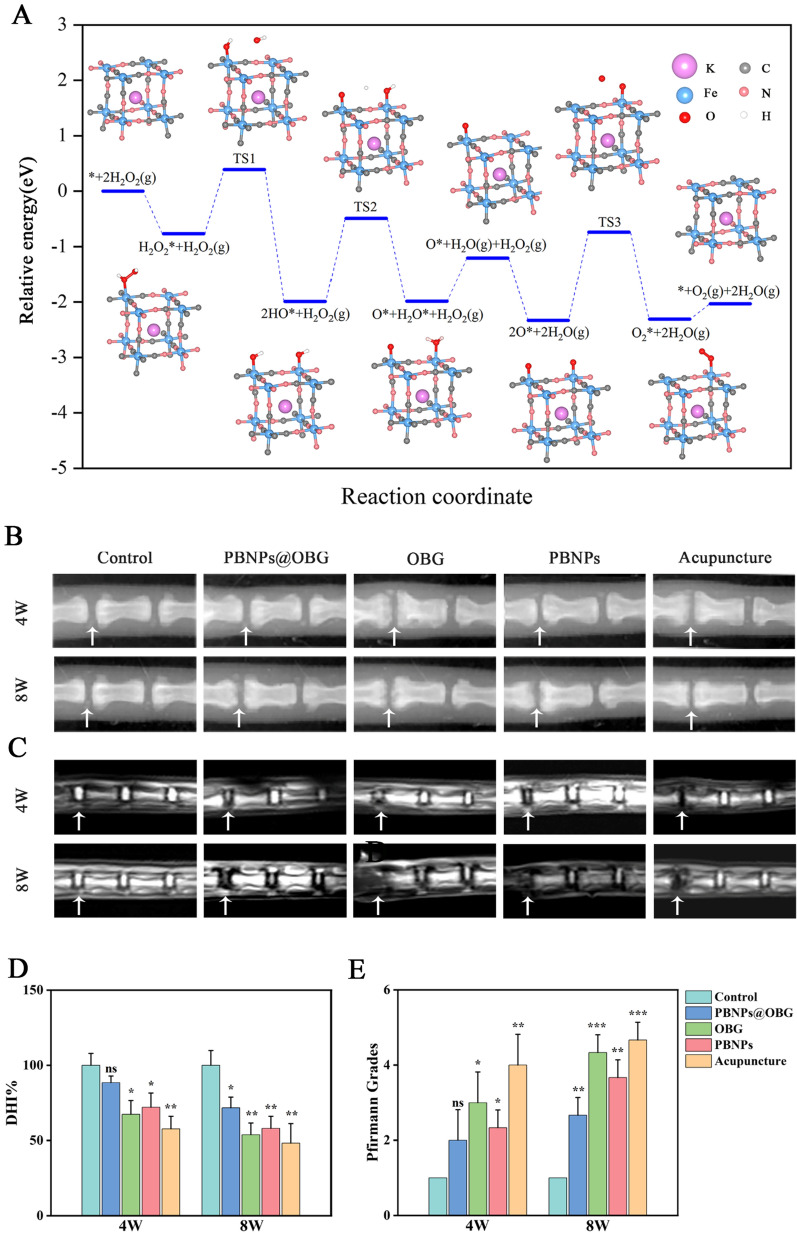
**A** DFT studies on the energy profile diagram of H2O2 decomposition into H2O and O2. Scale bar = 80 µm. **B**, **D** X-ray images and quantitative disc height index (DHI) analysis of rat coccygeal vertebrae at 4 and 8 weeks after operation (white arrows: position of the operation discs). **C**, **E** MRI scan images and Pfirrmann grade of rat tails at 4 and 8 weeks after disc surgery injected with different materials (white arrows). (n = 3, *P < 0.05, **P < 0.01, ***P < 0.001, *ns* not significant)

It was speculated that PBNPs could react with the most abundant endogenous ROS (H_2_O_2_) to produce H_2_O and O_2_ [62]. Density functional theory (DFT) calculations were carried out for an in-depth understanding of how H_2_O_2_ decomposes into H_2_O and O_2_. Geometrically optimized PBNPs observed from different angles were shown in Additional file 1: Figure S14). The first step was the adsorption of H_2_O_2_ onto PBNPs with adsorption energy of 0.76 eV. The H_2_O_2_* was decomposed into two HO* with an activation energy of 1.15 eV. The decomposition is an exothermic reaction that releases 1.22 eV. One H of OH* is transferred to another HO* to form H_2_O*, which is then desorbed with an energy of 0.79 eV. Another H_2_O_2_ molecule participates the reaction that generates an adsorbed O* and a H_2_O. The two O* form O_2_* with an activation energy of 1.59 eV. Then O_2_* desorbs from PBNPs, giving the final products to be H_2_O and O_2_. Yellow color in the charge density difference (CDD) implies charge gain and blue color implies loss of electric charge. All these figures are shown in the same iso-surface level at 0.003 e/(Bohr^3). Charge gain is observed in the bonding area between O atom and PBNPs substrate for H_2_O_2_, indicating an interaction between H_2_O_2_ and PBNPs. The area between O–O bond in H_2_O_2_ shows the depletion of charge, as indicated by blue color. Before H_2_O_2_ adsorption, Fe atom in PBNPs initially shows a charge loss of 0.89 e^−^ by Bader charge analysis. After H_2_O_2_ adsorbed to the Fe atom, the charge loss of Fe decreases to 0.86 e^−^, telling electrons are slightly transferred from H_2_O_2_ to Fe atom. The loss of electrons of H_2_O_2_ is consistent with CDD results that there is a deficiency of charge in O–O bond area, which can accelerate the breakage of O–O bond. The O–O bond is expected to break to form two OH due to the charge loss. The OH shows charge gain between O and PB, indicating strong interaction. From Bader charge analysis, the Fe atoms with OH adsorbed show an electron loss of 0.95 and 1.50 e^−^, respectively, much higher than the original state without OH adsorbed, which are 0.89 and 1.44 e^−^, respectively. The Bader analysis also shows the charge is transferred to OH, and consistent with CDD results. Within OH, there is a charge depletion between O–H bonds, which implies H can be broke facilely. One H_2_O will be formed when H in one of the OH transferred to another. With the loss of one H for OH, the electron gains of O changed from 1.00 to 0.50 e^−^. H contributes a lot to the accumulation of electrons in O atom clearly. Single O shows forceful interaction with PBNPs as illustrated by the large area of yellow color between single O and PBNPs. The electron loss of Fe from 0.89 to 1.04 e^−^ before and after O adsorption demonstrates that electrons are transferred to O. The two O atoms will merge together to form O_2_ molecule eventually. The tangy reciprocity between O atom and PBNPs is also reflected by the high activation energy as discussed earlier. O_2_ molecule shows a less charge gain between O and PBNPs compared with single O atom, which implies an easier desorption process. Bader charge manifests that the electron loss Fe for O_2_ adsorbed structure is similar to that without O_2_, further confirming the pregnable interaction between O_2_ and PBNPs, and an easier desorption of O_2_ molecule (Fig. 6A and Additional file 1: Figure S15).

Rat IVDD model was established to evaluate the in vivo effect of the PBNPs@OBG hydrogel. PBNPs@OBG, OBG, PBNPs, and PBS were injected into rat intervertebral disc, respectively, using a 26 G injector. After 4 and 8 weeks, rats were subjected to X-ray and Magnetic resonance imaging (MRI) (Fig. 6B, C). The disc height is the mirror of the ECM [63]. After 4- and 8 week post injection, the disc height index (DHI%) value of the Acupuncture group was prominently diminished. The DHI% value of the PBNPs@OBG group was similar to the Control group at 4 weeks, and slightly attenuation of DHI% was observed after 8 weeks post injection in the PBNPs@OBG group (Fig. 6D), indicating restraint of the degenerative degree of IVDD. In contrast, the decline in the DHI% of OBG and PBNPs group were more pronounced than the PBNPs@OBG group over time. MRI is a gold standard for diagnosis of IVDD. Healthy disc will appear white in T_2_-weighted MRI, which signifies higher water content. Conversely, the degenerative discs turned black due to the dehydration of the tissues in T_2_-weighted images. The degree of disc degeneration was assessed by Pfirrmann MRI grade scores according to previous reports [64]. Eventual outcome of MRI analysis was in accordance with estimate of DHI% (Fig. 6E).

**Fig. 7:**
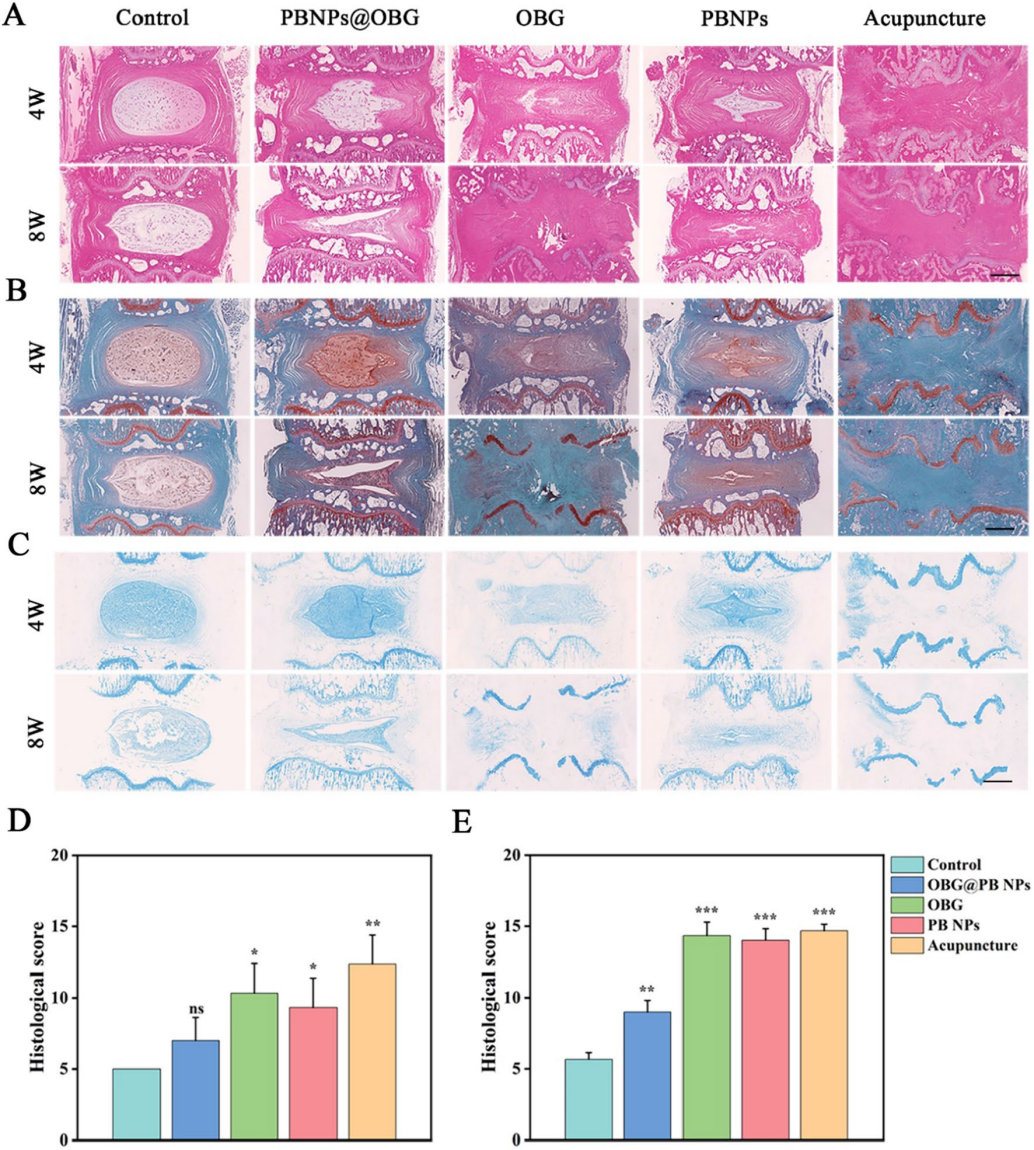
Histological images of animal experiments. **A** Representative images of H&E staining. Scale bar = 800 µm. **B** Representative pictures of Safranin-O/Fast staining at different timepoints. Scale bar = 800 µm. **C** Representative images of Alcian Blue staining at 4 and 8 weeks after operation. Scale bar = 800 µm. **D**, **E** Histological grades at week 4 and week 8 post-surgery in five groups (n = 3, *P < 0.05, **P < 0.01, ***P < 0.001, *ns* not significant)

Hematoxylin and eosin (H&E) staining was used to investigate fibrous tissue, margins, and NP morphology. From 4 to 8 weeks, in the Acupuncture group, the NP cells were replaced by disorganized hypocellular fibrocartilaginous tissue, and at the meantime the AF structure was wrecked along with atrophied NP volume. 8 weeks after operation, the AF structure was destructed more seriously compared with 4 weeks. In the early stage (4 W), the PBNPs@OBG group showed more intact composition than the OBG and PBNPs group, merely manifested as mildly reduction of NP cells. NP in the OBG and PBNPs group collapsed gradually and the borders were blurred and indistinct. After 8 weeks, regions of the NP were replaced by the annulus fibrosus in large part, along with worsened NP status. Alternatively, the PBNPs@OBG group displayed a much more integrated morphological arrangement (Fig. 7A). Safranin-O/Fast Green staining revealed that the proteoglycans of the PBNPs@OBG group were better preserved, while for other groups (OBG, PBNPs, and Acupuncture), the NP was superseded by collagen ultimately (Fig. 7B), which was consistent with the observation of Alcian Blue staining (Fig. 7C). The histological score was computed according to previous research [65]. At 4 weeks after operation, the histological score of the PBNPs@OBG group gained ground on the Control group (Fig. 7D). The score of the PBNPs@OBG group presented a much more tardigrade progression than other groups (OBG, PBNPs, and Acupuncture) in the long run (8 weeks) (Fig. 7E). Immunofluorescence staining shows that the expressions of aggrecan and collagen II in the PBNPs@OBG group were up-regulated compared to OBG, PBNPs, and Acupuncture groups, while the expressions of MMP3 and MMP13 in the PBNPs@OBG group were down-regulated in NP region at 8 weeks after surgical procedures (Additional file 1: Figure S16).

### Biocompatibility and biosafety evaluation

The cytotoxicity was determined by CCK-8 assay on human NP cells using the exudate of the OBG and PBNPs@OBG hydrogel (Additional file 1: Figure S17). No apparent biological toxicity was examined in all groups after 1, 3, and 5 days. Living/dead cell staining revealed that almost no dead cells were observed after 1, 3, and 5 days of incubation, which was consistent with the outcome of CCK-8 assay. All kinds of blood routine examinations including white blood cells, red blood cells, and platelets (Additional file 1: Figure S18 − S23), standard blood biochemical (Additional file 1: Figure S24−S25), and histological screening (Additional file 1: Figure S26−S27) were gauged after administration of various materials after 4 and 8 weeks. These data implied that no remarkable acute, chronic pathological toxicity, and untoward reaction were observed in the course of 8 weeks. This emphasized that the hydrogel was of good biosafety to be applied in tissue engineering area.

## Discussion

It is still a challenge to search appropriate delivery strategies to alleviate IVDD. Gan et al*.* come up with an interpenetrating network-strengthened hydrogel for NP regeneration with toughness and cytocompatibility [66], while they did not pay attention to the importance of antimicrobial effect for minimally invasive injection. Zhou et al*.* also treated IVDD from the perspective of antioxidation [67], however, pure liquid injection lacks mechanical properties, which is indispensable for loading bearing tissues. Gullbrand et al*.* developed tissue-engineered, endplate-modified disc-like angle ply structures (eDAPS) for disc replacement [68]. Nevertheless, implantation of eDAPS requires surgery which is not as simple as minimally invasive injection. Our multifunctional hydrogel conquers above defects and demonstrates that the hydrogel can be applied as a promising delivery strategies for IVDD treatment.

## Conclusion

In summary, we prepared a novel dual-dynamic-bond cross-linked injectable self-healing hydrogel with antibacterial and antioxidant properties as well as excellent mechanical characters for IVDD repair. The smart PBNPs@OBG hydrogel could be injected into the degenerated intervertebral disc via minimal invasive method and provide appropriate mechanical support. By taking advantages of the sustained release of PBNPs from the PBNPs@OBG hydrogel, long time retention of PBNPs in disc and effective antioxidant therapy could be achieved. Profit from versatile integration of hydrogels, as-fabricated PBNPs@OBG hydrogel could protect NP cells against ROS overproduction, restore the disc height, attenuate the decrease of water content, and reverse the IVDD disordered microenvironment. We believe that the smart multifunctional hydrogel could serve as a promising candidate for IVDD treatment.

## Supplementary Information

**Additional file 1: Figure S1.** Abridged general view of PBNPs formation. **Figure S2.** Dynamic light scattering (DLS) of the PBNPs. **Figure S3.** Thermogravimetry-differential scanning calorimetry (TG-DSC) analyses of the PBNPs. **Figure S4.** Adsorption isotherms of the PBNPs, demonstrating its mesoporous structure. **Figure S5.** Photographs of OBG hydrogel formation. **Figure S6.** SEM images of the OBG and PBNPs@OBG. Scale bar = 100 µm. **Figure S7.** EDS mapping images of the PBNPs@OBG. Scale bar = 100 µm. **Figure S8.** In vitro degradation percentages of the PBNPs@OBG in vitro, in PBS with different pH values of 7.4 and 6.5 at 37 °C. **Figure S9.** Photograph of the OBG and PBNPs@OBG in compression tests. **Figure S10.** **A** Weight lifting ability for the glass adhered by the PBNPs@OBG hydrogel. **B** Adhesiveness strengths of the OBG and PBNPs@OBG (*n* = 3, *ns* not significant). **Figure S11.** H_2_O_2_ decomposed into H_2_O and O_2_ in the presence of PBNPs within 10 min. **Figure S12.** JC-1 staining quantified as the ratios of Red/Green fluorescence intensities. (*n *= 3, **P < 0.01 versus H_2_O_2_ alone.). **Figure S13.** **A**–**B** Effect of the PBNPs@OBG on NPs apoptosis after H_2_O_2_ treatment by annexin V-FITC and propidium iodide staining. (*n *= 3, **P < 0.01, ***P < 0.001). **Figure S14.** Geometrically optimized PBNPs observed from different angles. **Figure S15.** Charge density difference of different species adsorbed on the PBNPs substrates. The yellow color represents charge accumulation, while green color is the charge loses. **Figure S16.** Immunofluorescence staining of aggrecan, collagen II, MMP3, and MMP13 in NP tissues at 8 weeks after injection. Scale bar = 50 µm. **Figure S17.** **A** Living/dead staining images of NP cells *in vitro*. Scale bar = 200 µm. **B** CCK-8 assay of NP cells after being treated with different materials. **Figure S18.** Blood routine examination results of WBC at 4 weeks. 1. Control; 2. PBNPs@OBG; 3. OBG; 4. OBG; 5. Acupuncture (*n* = 3, *ns* not significant). **Figure S19.** Blood routine examination results of WBC at 8 weeks. 1. Control; 2. PBNPs@OBG; 3. OBG; 4. OBG; 5. Acupuncture (n = 3, *ns* not significant). **Figure S20.** Blood routine examination results of RBC at 4 weeks. 1. Control; 2. PBNPs@OBG; 3. OBG; 4. OBG; 5. Acupuncture (n = 3, *ns* not significant). **Figure S21.** Blood routine examination results of RBC at 8 weeks. 1. Control; 2. PBNPs@OBG; 3. OBG; 4. OBG; 5. Acupuncture (*n* = 3, *ns* not significant). **Figure S22.** Blood routine examination results of platelets at 4 weeks. 1. Control; 2. PBNPs@OBG; 3. OBG; 4. OBG; 5. Acupuncture (*n* = 3, *ns* not significant). **Figure S23.** Blood routine examination results of platelets at 8 weeks. 1. Control; 2. PBNPs@OBG; 3. OBG; 4. OBG; 5. Acupuncture (*n* = 3, ns: not significant). **Figure S24.** Standard blood biochemical examination at 4 weeks. 1. Control; 2. PBNPs@OBG; 3. OBG; 4. OBG; 5. Acupuncture (*n* = 3, **P < 0.01, *ns* not significant). **Figure S25.** Standard blood biochemical examination at 8 weeks. 1. Control; 2. PBNPs@OBG; 3. OBG; 4. OBG; 5. Acupuncture (*n* = 3, *ns* not significant). **Figure S26.** H&E staining of heart, liver, spleen, lung, and kidney at 4 weeks after operation. Scale bar = 100 µm. **Figure S27.** Histological screening at 8 weeks after operation. Scale bar = 100 µm. **Table S1.** Primer used for Qpcr.

**Additional file 2. **Video of OBG hydrogel formation.
