# Supplementary material for: Dual-dynamic-bond cross-linked injectable hydrogel of multifunction for intervertebral disc degeneration therapy
Source: J Nanobiotechnology. 2022 Oct 1;20:433. doi: 10.1186/s12951-022-01633-0 (PMC9526989; doi:10.1186/s12951-022-01633-0)
Supplement: Supplementary file 1 — Additional file 1: Figure S1. Abridged general view of PBNPs formation. Figure S2. Dynamic light scattering (DLS) of the PBNPs. Figure S3. Thermogravimetry-differential scanning calorimetry (TG-DSC) analyses of the PBNPs. Figure S4. Adsorption isotherms of the PBNPs, demonstrating its mesoporous structure. Figure S5. Photographs of OBG hydrogel formation. Figure S6. SEM images of the OBG and PBNPs@OBG. Scale bar = 100 µm. Figure S7. EDS mapping images of the PBNPs@OBG. Scale bar = 100 µm. Figure S8. In vitro degradation percentages of the PBNPs@OBG in vitro, in PBS with different pH values of 7.4 and 6.5 at 37 °C. Figure S9. Photograph of the OBG and PBNPs@OBG in compression tests. Figure S10. A Weight lifting ability for the glass adhered by the PBNPs@OBG hydrogel. B Adhesiveness strengths of the OBG and PBNPs@OBG (n = 3, ns not significant). Figure S11. H2O2 decomposed into H2O and O2 in the presence of PBNPs within 10 min. Figure S12. JC-1 staining quantified as the ratios of Red/Green fluorescence intensities. (n = 3, **P < 0.01 versus H2O2 alone.). Figure S13. A–B Effect of the PBNPs@OBG on NPs apoptosis after H2O2 treatment by annexin V-FITC and propidium iodide staining. (n = 3, **P < 0.01, ***P < 0.001). Figure S14. Geometrically optimized PBNPs observed from different angles. Figure S15. Charge density difference of different species adsorbed on the PBNPs substrates. The yellow color represents charge accumulation, while green color is the charge loses. Figure S16. Immunofluorescence staining of aggrecan, collagen II, MMP3, and MMP13 in NP tissues at 8 weeks after injection. Scale bar = 50 µm. Figure S17. A Living/dead staining images of NP cells in vitro. Scale bar = 200 µm. B CCK-8 assay of NP cells after being treated with different materials. Figure S18. Blood routine examination results of WBC at 4 weeks. 1. Control; 2. PBNPs@OBG; 3. OBG; 4. OBG; 5. Acupuncture (n = 3, ns not significant). Figure S19. Blood routine examination results of WBC at 8 weeks [file 12951_2022_1633_MOESM1_ESM.docx]

**Supporting Information**

**Dual-Dynamic-Bond Cross-Linked Injectable Hydrogel of Multifunction for Intervertebral Disc Degeneration Therapy**

Linjun Yang ^1†^, Congcong Yu ^1†^, Xuhui Fan ^2^, Tianni Zeng ^3^, Wentao Yang ^1^, Jiechao Xia ^1^, Jianle Wang ^1^, Litao Yao ^4^, Chuan Hu ^1^, Yang Jin ^1^, Yutao Zhu ^1^, Jiaxin Chen ^1^, Zhijun Hu ^1*^

^1^ Department of Orthopaedic Surgery, Sir Run Run Shaw Hospital, Zhejiang University School of Medicine, Key Laboratory of Musculoskeletal System Degeneration, Regeneration Translational Research of Zhejiang Province, 3 East Qing Chun Road, Hangzhou 310002, P. R. China.

^2^ Department of Radiology, Shanghai General Hospital, Shanghai Jiao Tong University School of Medicine, 100 Haining Road, Shanghai 200080, P. R. China.

^3^ Department of Oncology, Hangzhou TCM hospital Affiliated to Zhejiang Chinese Medical University, 453 Tiyuchang Road, Hangzhou 310007, P. R. China.

^4^ Department of Dentistry, Sir Run Run Shaw Hospital, School of Medicine, Zhejiang University, 3 East Qing Chun Road, Hangzhou 310002, P. R. China.

^†^ Linjun Yang and Congcong Yu contribute equally to this work.

* To whom correspondence should be addressed, [hzjspine@zju.edu.cn](mailto:hzjspine@zju.edu.cn) (Z. Hu).

**Experimental Section**

**Rheological Studies**

Strain amplitude sweep test (*γ* = 1%−1000%) was conducted to get the intersection point. Frequency dependent rheological measurement was performed with a strain rate of 1%, 37 °C. Step-strain test was switched between a low strain (*γ* = 1%) and a high strain (*γ* = 400%) with an angular frequency of 10 rad s^−1^, 37 °C. Shear-thinning behavior of the hydrogel was carried out at 37 °C, with a shear rate range from 0.01 to 100 s^−1^. Thermoresponsive reversible property was conducted by dynamic temperature sweep tests, and the temperature varied between 37 and 20 ℃, and a temperature change of 10−40 °C was carried out.

***In Vitro* Swelling Test**

The dry weight of freeze-dried hydrogels was recorded as *W*_0_. Subsequently, hydrogels were soaked into PBS solution at 37 ℃, and the moist hydrogel was weighted at a certain time and recorded as *W*_t_. The swelling ratio was calculated as follows:

Swelling ratio (%) = (*W*_t_ − *W*_0_)/*W*_0_.

***In Vitro* Degradation Test**

The dry weight of freeze-dried hydrogel was recorded as *W*_0_, Subsequently, hydrogels were soaked into PBS solution at 37 ℃ for 24 h to achieve equilibrium swelling state, and hydrogels were incubated in PBS solution and weighted at a certain time, which was recorded as *W*_t_. The degradation level was calculated as follows:

Relative weight (%) = *W*_t_ */ W*_0_ × 100.

**Mechanical Property Test**

The tensile and compression tests were conducted by a dynamic mechanical analysis (DMA, Q800, America). The samples were molded into cylindrical specimens (diameter = 2 cm and height = 5 mm) for tensile tests. Samples molded into cylindrical specimens (diameter = 8 mm and height = 8 mm) were used for compression tests. Both the tests were carried out at a speed of 10 mm/min at 37 ℃.

**Adhesion Measurements**

The prepared hydrogels were applied homogeneously between two pieces of porcine skin, which was 1 cm in width and 2 cm in length. The contact area was maintained at 1 cm × 1 cm. After gelation for 12 h, DMA with a speed of 1 mm min^−1^ was used to perform lap-shear test.

**Cell Isolation and Culture**

Human nucleus pulposus cells were isolated from patient specimens undertaking lumbar discectomy or surgery due to thoracolumbar fracture or scoliosis. NP tissues were obtained from the first bite of tissues by a laminectomy rongeur without any damaging the vertebral endplate, which was collected from discarded surgical waste. Informed consent was obtained from each patient, and our study protocol was approved by the Ethical Review Board of Sir Run Shaw Hospital (Zhejiang, China). Human NP tissues were cut into small pieces and washed with PBS several times, then treated with 0.2% collagenase type II at 37 ℃ for 4 h, followed by filtered through a 40 mm pore size mesh. The nucleus pulposus cells were maintained in DMEM and Ham’s F-12 medium (DMEM/F12) supplemented with 10% FBS DMEM and 10% FBS at 37 ℃ in an atmosphere of 5% CO_2_.

***In Vitro* Antibacterial Properties**

The antibacterial ability of the hydrogels was tested by Kirby-Bauer disk diffusion test. First, the density of Staphylococcus aureus and Escherichia coli were adjusted to 10^6^ CFU mL^−1^. Second, the *S. aureus* and *E. coli* were planted on the agar plates surface using a cotton swab. Finally, the two hydrogel samples were placed in the center of agar plates, respectively, which *co*-cultured with *S. aureus* and *E. coli* for 12 or 36 h at 37 °C with 5% CO_2_. Measuring the diameter of the antibacterial cycle after *co*-culturing and calculating.

**Intracellular ROS (H_2_O_2_) depletion**

NP cells were seeded in a 12-well plate at a density of 5 × 10^4^ cells *per* well for 24 h. Cells were treated with 10 μM DCFH-DA and incubated for 30 minutes. Then the NP cells were disposed with DMEM (10% FBS) solution containing extracts of OBG@PBNPs hydrogel with H_2_O_2_ (500 µM) for 30 minutes. Cells treated with H_2_O_2_ (500 µM) or no treatment were used as the positive control and negative control. Then the cell pictures were captured by CLSM or determined by flow cytometry. Then, cell apoptosis was determined by Annexin V-FITC Apoptosis Detection Kit.

**JC-1 Staining**

NP cells were seeded in a 12-well plate at a density of 5 × 10^4^ cells *per* well for 24 h. The cells were fixed with 4% PFA for 20 minutes. The mitochondria were stained with JC-1 dye according to the manufacturer protocol. The nucleus was stained with DAPI, and then the cells were imaged by CLSM.

**Gene Expression**

Total RNA was extracted using the Trizol reagent after being washed three times with PBS. The RNA quality was detected by NanoDrop, the total RNA was reverse transcribed into cDNA in a 10-μL reaction system. The qRT-PCR assay was performed using SYBR Green mix from Takara on Light Cycler 96. Relative gene quantitation was determined by the 2^-ΔΔCT^ method. The primer sequences for COL II, MMP3, MMP13, SOX9, and β-actin are listed in Table S1 (Supporting Information).

**Western Blot**

Treated NPs were isolated using radioimmunoprecipitation assay buffer with phenylmethanesulfonyl fluoride. Protein concentration was measured using a BCA protein assay kit. Proteins were separated on sodium dodecyl sulfate-polyacrylamide gels and transferred onto polyvinylidene difluoride membranes. The membranes were blocked in 5% skim milk, and then incubated with primary antibodies specific for COL II, MMP3, MMP13, SOX9, and β-actin overnight at 4 ℃. Then incubation with the respective secondary antibodies at room temperature for 2 h. The bands were detected by ECL reagent, followed by visualized using the LAS-4000 Science Imaging System.

**Cell Proliferation Assay**

NP cells were seeded in 96-well plate at a density of 5 × 10^3^ cells/well for 24 h. The 200 μL medium was replaced by the extracts, and the former culture medium was replaced by 100 μL of fresh DMEM containing 10% CCK-8 (*V/V*) and incubated at 37 °C for 2 h. The absorbance of the medium at 450 nm was measured with a plate reader.

**Cell Viability Assay**

NP cells were seeded in 24-well plate at a density of 5 × 10^4^ cells/well for 24 h. The culture medium was replaced by the extracts, and the samples were further cultured in an atmosphere of 5% CO_2_ for 1, 3, or 5 days at 37 °C and assessed by Live/Dead kit.

***In Vivo* Retention of PBNPs**

PBNPs were dispersed in 5 mL PBS and 5 mg Cy5 with stir for several time, then centrifuged and washed with PBS for several times, and then Cy5 labeled PBNPs was obtained. 20 μL Cy5-PBNPs and Cy5-PBNPs@OBG were injected into Sprague-Dawley rats C6-7 intervertebral disc with 26 G needle, respectively. All rats underwent *in vivo* imaging used IVIS spectral at predetermined time points. Captured images were analyzed *via* the Living Image software.

**DFT Calculations**

Spin-polarized DFT calculations were performed using the Vienna ab initio simulation package [1, 2]. The generalized gradient approximation proposed by Perdew, Burke, and Ernzerhof (GGA-PBE) was selected for the exchange-correlation potential [3]. The pseudo-potential was described by the projector-augmented-wave method [4]. Geometry optimization was performed until the Hellmann-Feynman force on each atom was smaller than 0.04 eV·Å^−1^. The energy criterion was set to 10^−6^ eV in iterative solution of the Kohn-Sham equation. Prussian blue (001) slab with 6 atomic layers and 12 Å vacuum was built. Moreover, nudged elastic band method [5, 6] was used to search for transition state structures.

**Radiological Evaluation**

X-ray was performed at 4 and 8 weeks after surgery. Disc height was measured using Image J and DHI was calculated as described in the previous method. MRI parameters were set as follows:^7^ slew rate at 150 mT m^−1^ ms^−1^, and gradient field intensity of 30 mT m^−1^. Parameters for spinecho sequence were T_2_WI/TR, TE = 3500 ms/120 ms, scan matrix: 256 × 256, reconstruction matrix: 512 × 512, FOV (mm) = 100.00, RFOV (%) = 100.00, slice thickness = 3 mm, and scan resolution = 0.3 mm.


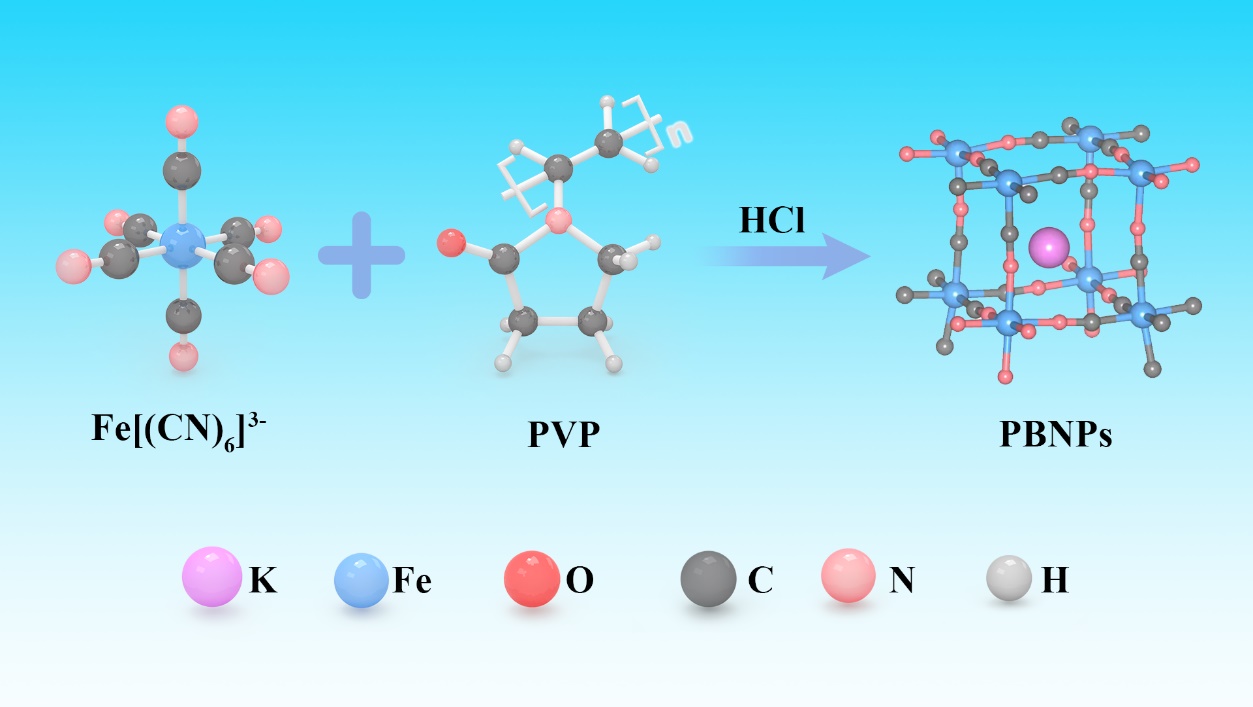


**Additonal file 1: Figure S1.** Abridged general view of PBNPs formation.





**Additonal file 1: Figure S2.** Dynamic light scattering (DLS) of the PBNPs.





**Additonal file 1: Figure S3.** Thermogravimetry-differential scanning calorimetry (TG-DSC) analyses of the PBNPs.





**Additonal file 1: Figure S4.** Adsorption isotherms of the PBNPs, demonstrating its mesoporous structure.


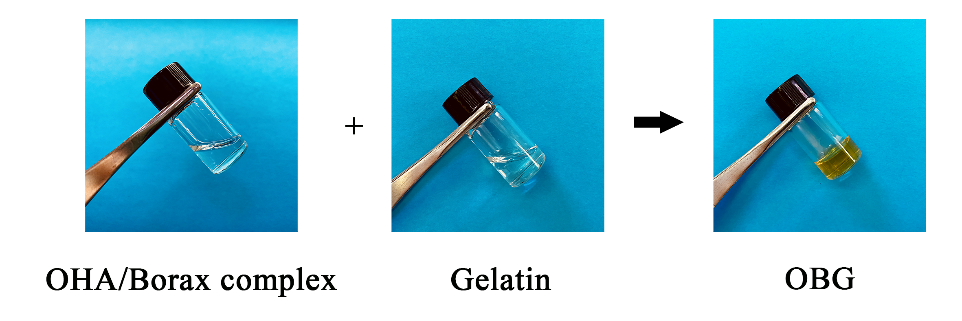


**Additonal file 1: Figure S5.** Photographs of OBG hydrogel formation.


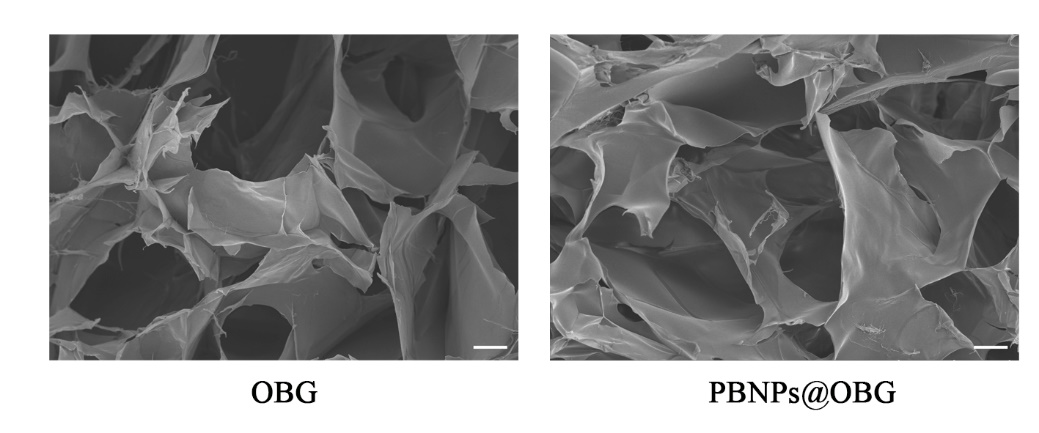


**Additonal file 1: Figure S6.** SEM images of the OBG and PBNPs@OBG. Scale bar = 100 µm.


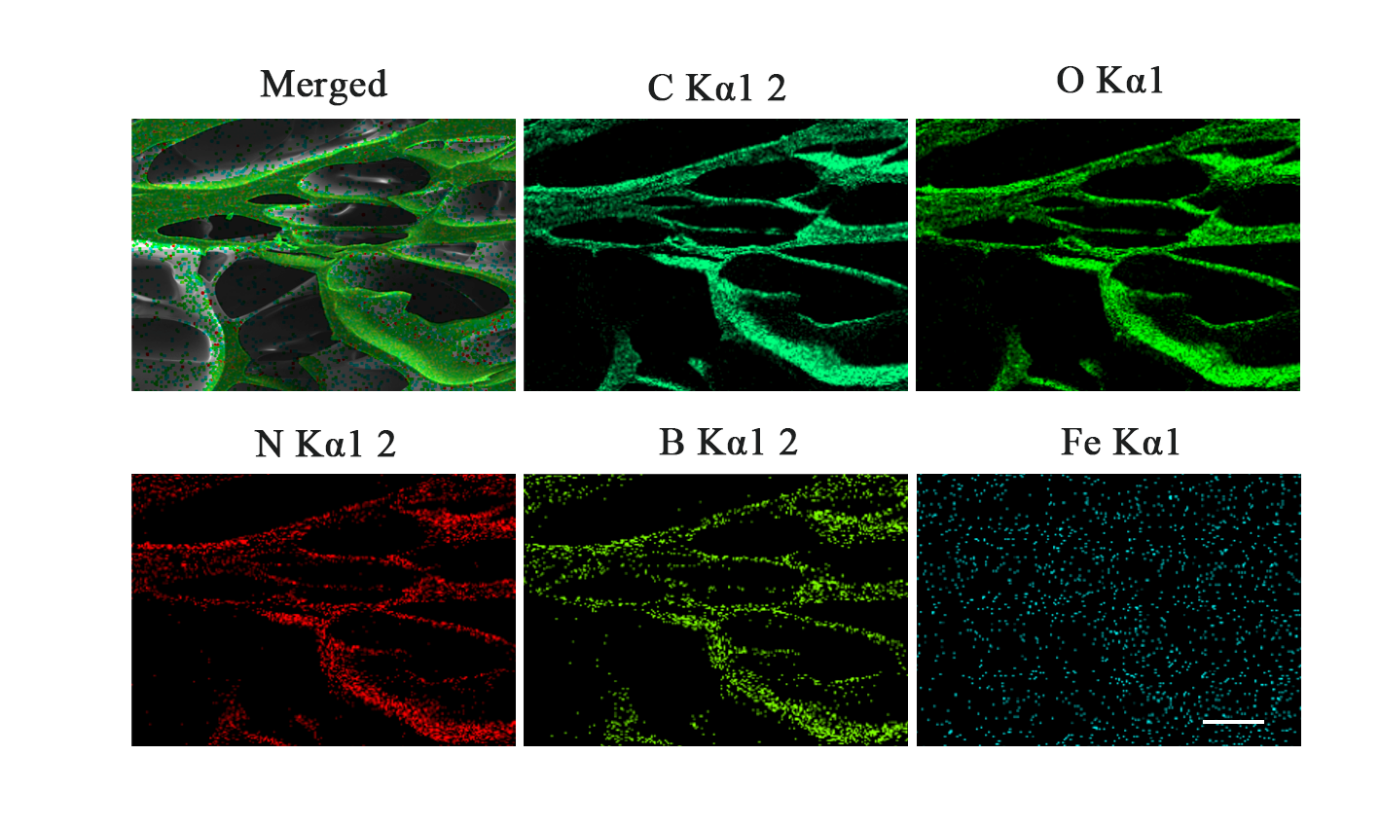


**Additonal file 1: Figure S7.** EDS mapping images of the PBNPs@OBG. Scale bar = 100 µm.





**Additonal file 1: Figure S8.** *In vitro* degradation percentages of the PBNPs@OBG in vitro, in PBS with different pH values of 7.4 and 6.5 at 37 ℃.


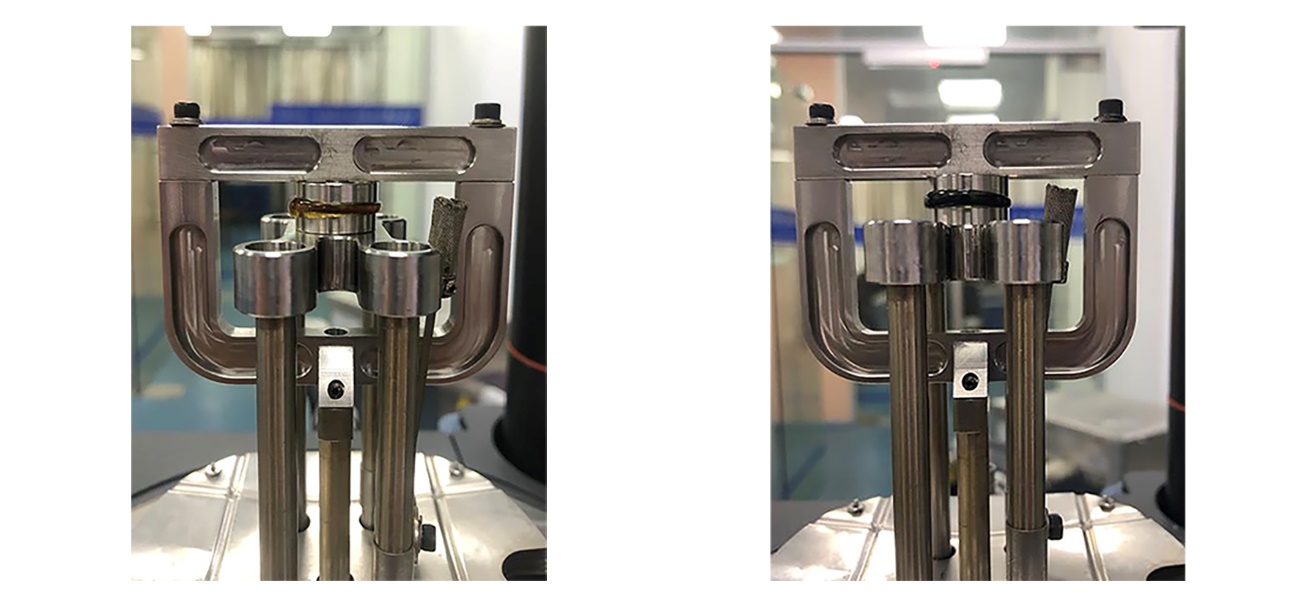


**Additonal file 1: Figure S9.** Photograph of the OBG and PBNPs@OBG in compression tests.


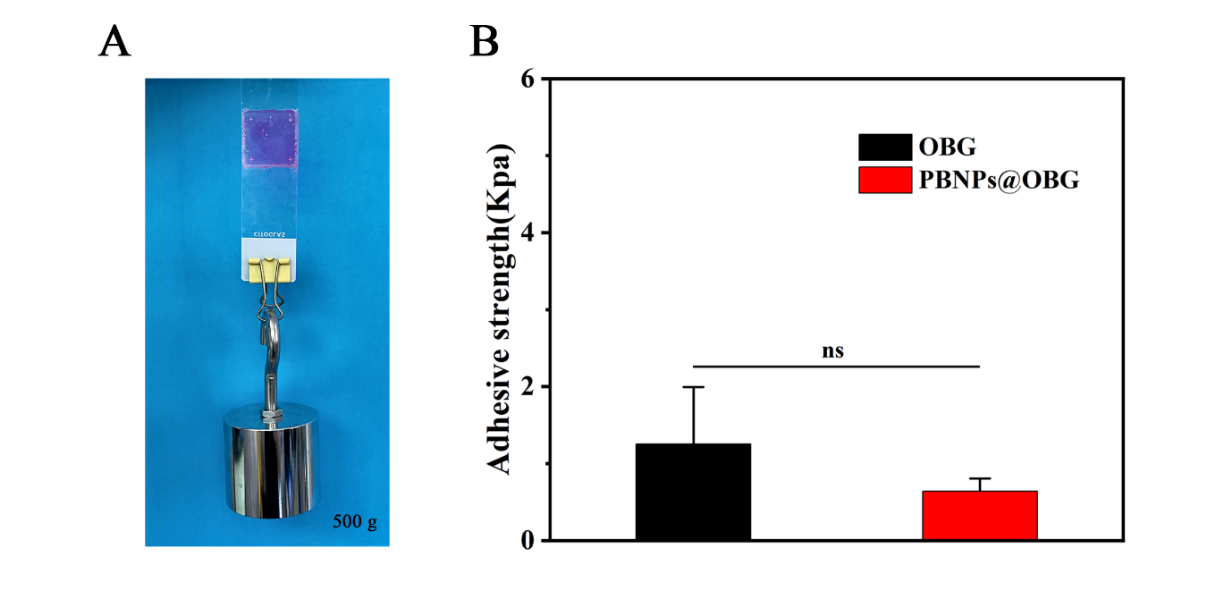


**Additonal file 1: Figure S10.** (A) Weight lifting ability for the glass adhered by the PBNPs@OBG hydrogel. (B) Adhesiveness strengths of the OBG and PBNPs@OBG (*n* = 3, ns: not significant).


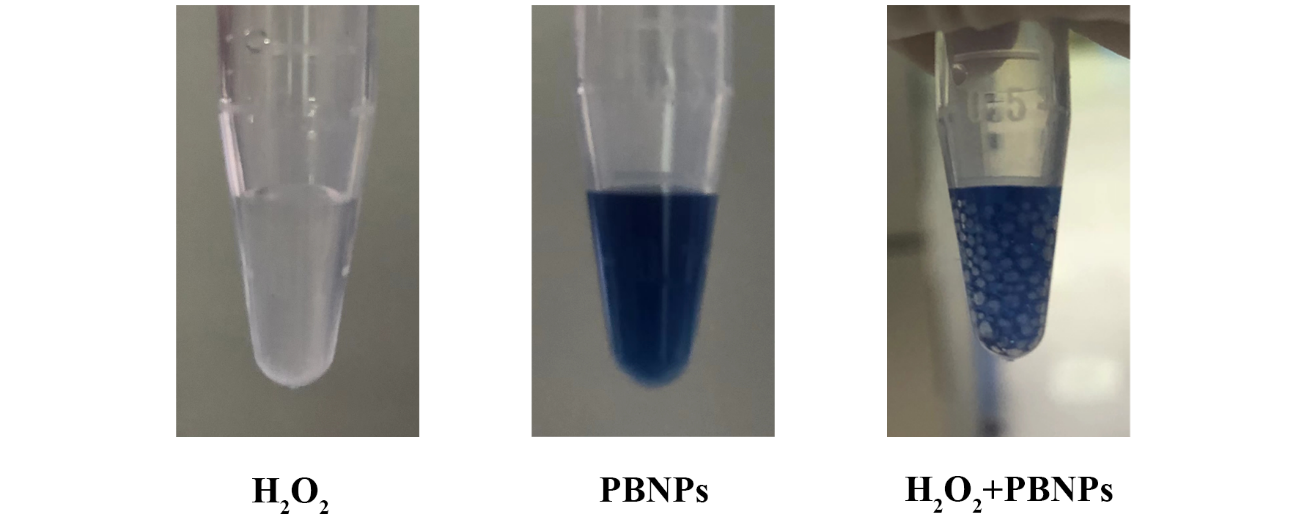


**Additonal file 1: Figure S11.** H_2_O_2_ decomposed into H_2_O and O_2_ in the presence of PBNPs within 10 min.





**Additonal file 1: Figure S12.** JC-1 staining quantified as the ratios of Red/Green fluorescence intensities. (*n* = 3, ***P* < 0.01 versus H_2_O_2_ alone.)


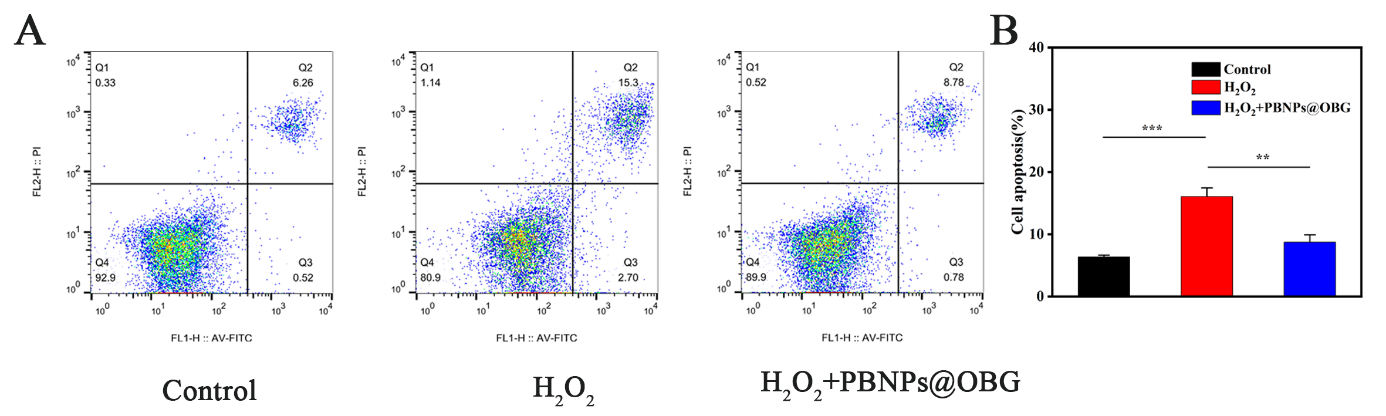


**Additonal file 1: Figure S13.** (A-B) Effect of the PBNPs@OBG on NPs apoptosis after H_2_O_2_ treatment by annexin V-FITC and propidium iodide staining. (*n* = 3, ***P* < 0.01, ****P* < 0.001).


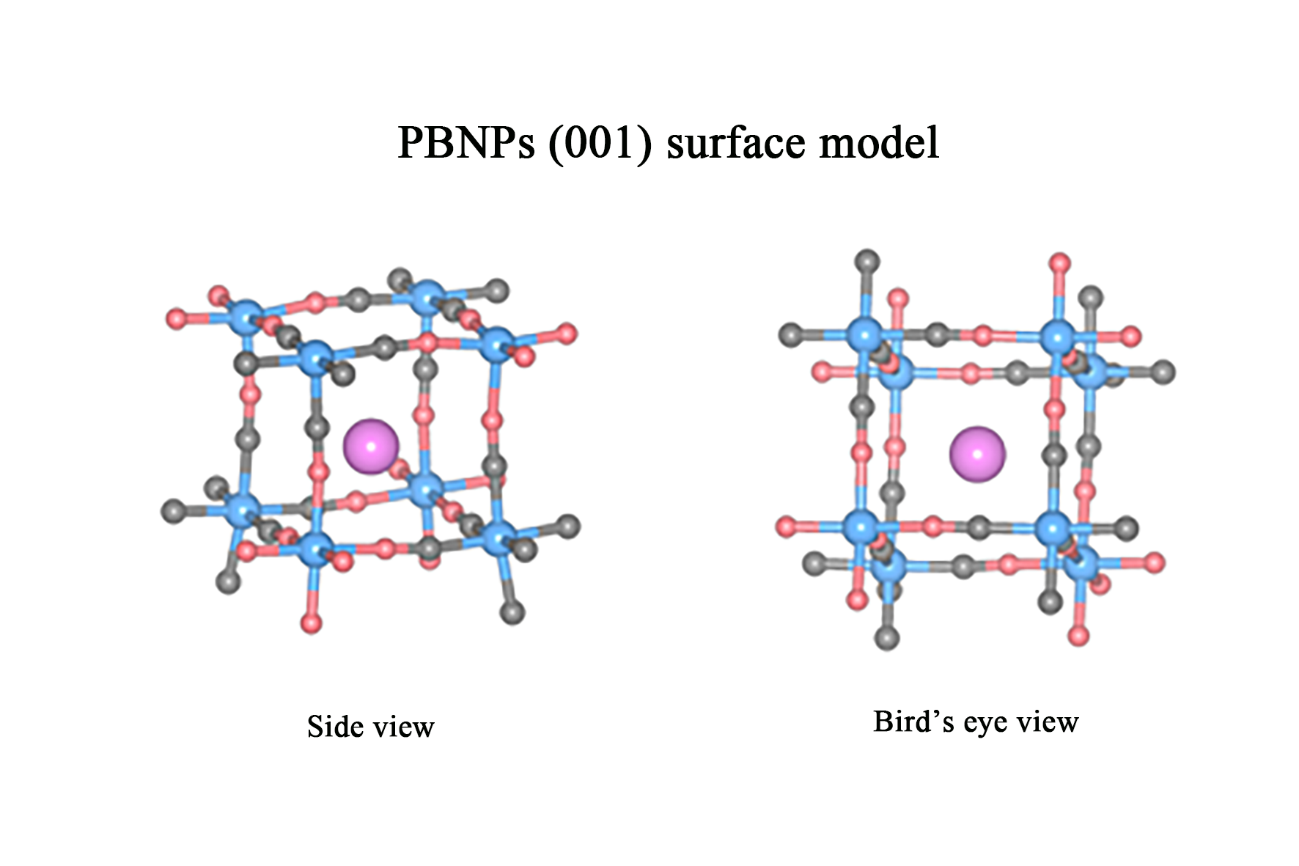


**Additonal file 1: Figure S14.** Geometrically optimized PBNPs observed from different angles.

**
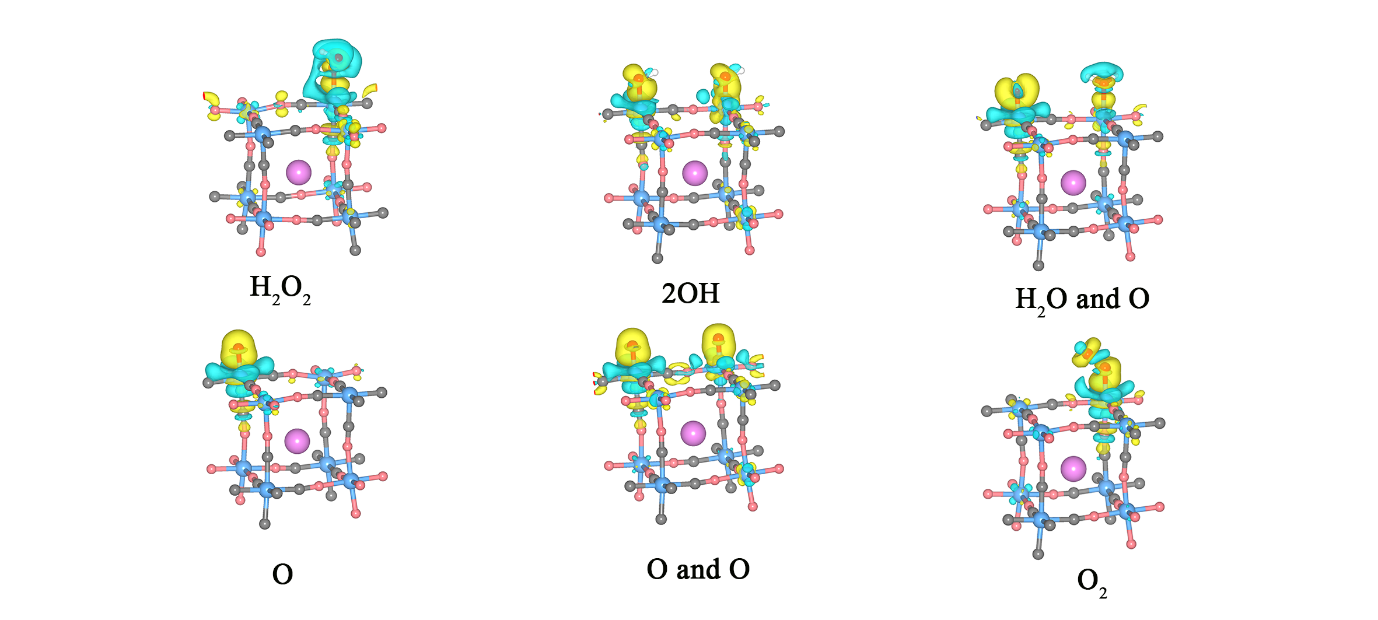
**

**Additonal file 1: Figure S15.** Charge density difference of different species adsorbed on the PBNPs substrates. The yellow color represents charge accumulation, while green color is the charge loses.


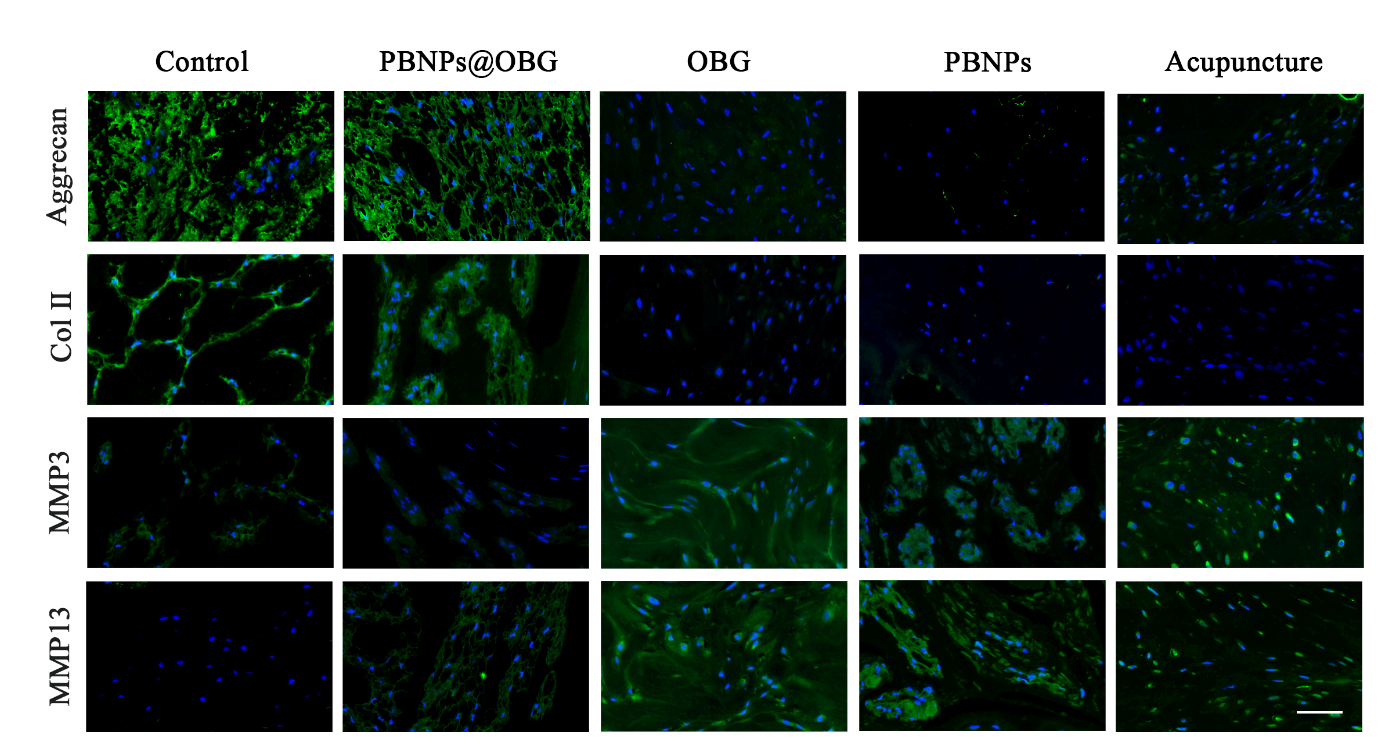


**Additonal file 1: Figure S16.** Immunofluorescence staining of aggrecan, collagen Ⅱ, MMP3, and MMP13 in NP tissues at 8 weeks after injection. Scale bar = 50 µm.


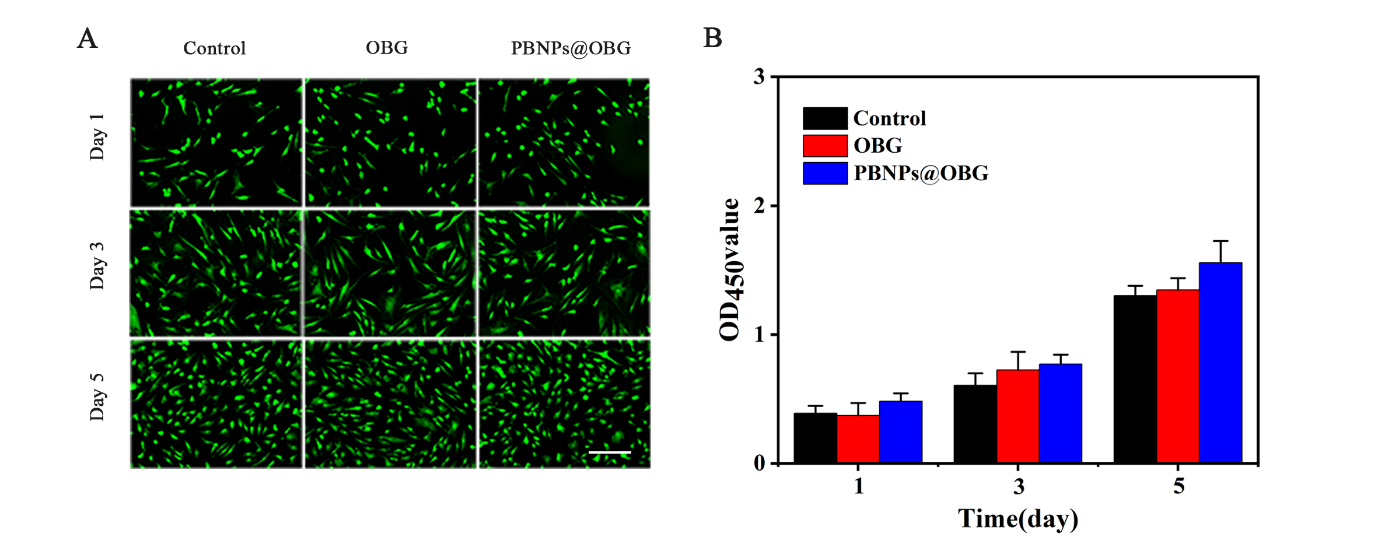


**Additonal file 1: Figure S17.** (A) Living/dead staining images of NP cells *in vitro*. Scale bar = 200 µm. (B) CCK-8 assay of NP cells after being treated with different materials.


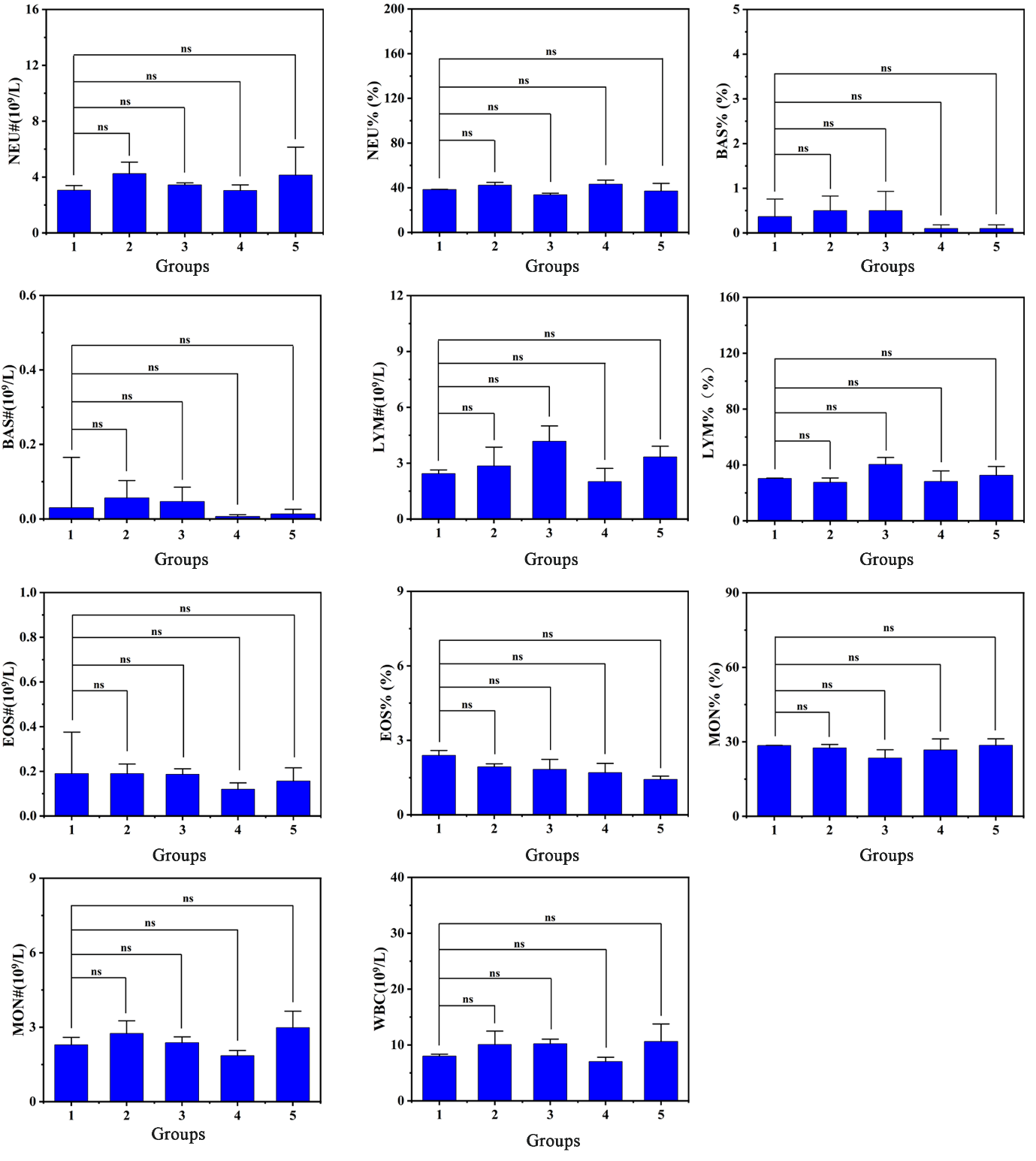


**Additonal file 1: Figure S18.** Blood routine examination results of WBC at 4 weeks. 1. Control; [2. PBNPs@OBG](mailto:2.OBG@PB); 3. OBG; 4. OBG; 5. Acupuncture (*n* = 3, ns: not significant).


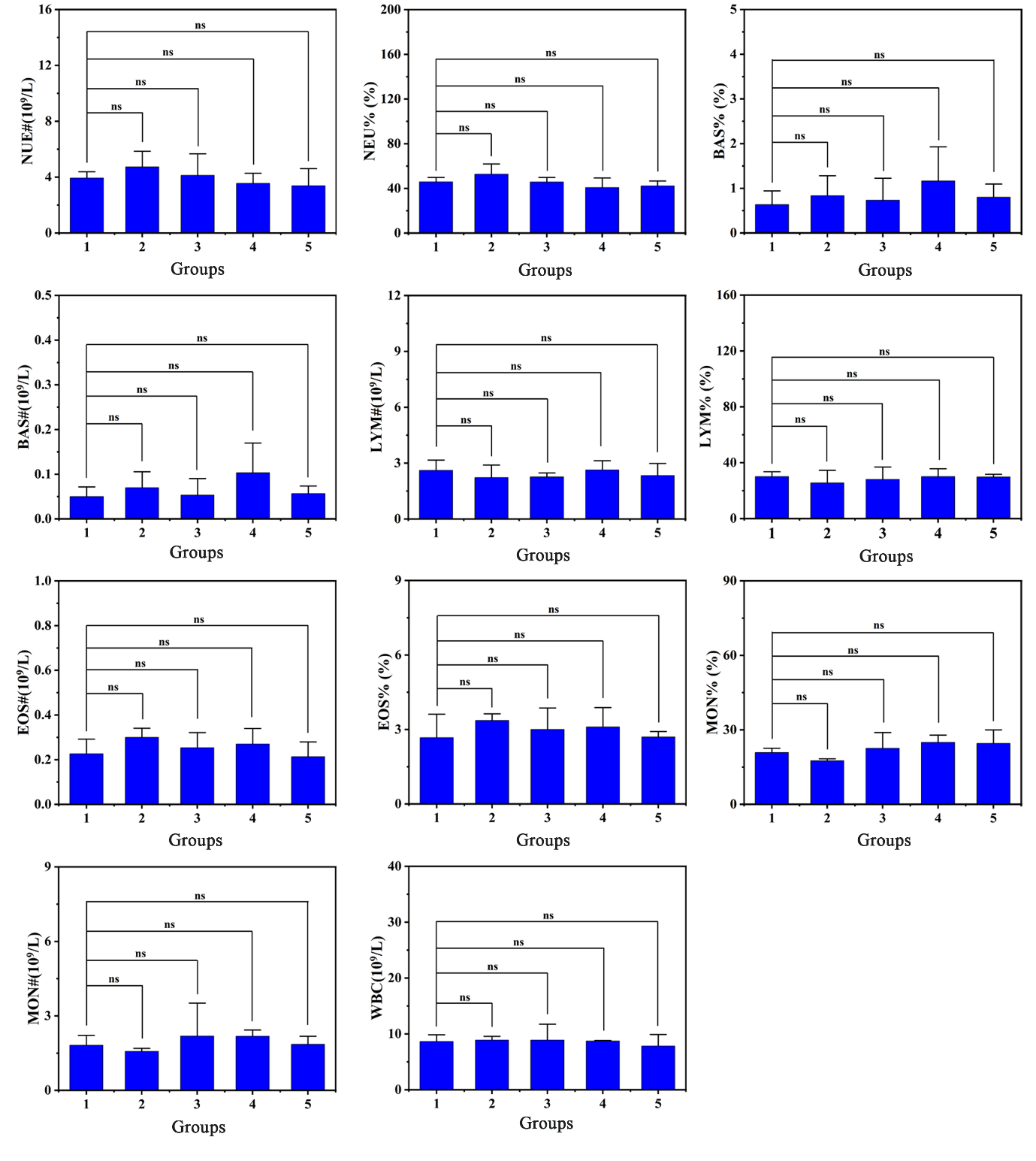


**Additonal file 1: Figure S19.** Blood routine examination results of WBC at 8 weeks. 1. Control; [2. PBNPs@OBG](mailto:2.OBG@PB); 3. OBG; 4. OBG; 5. Acupuncture (*n* = 3, ns: not significant).


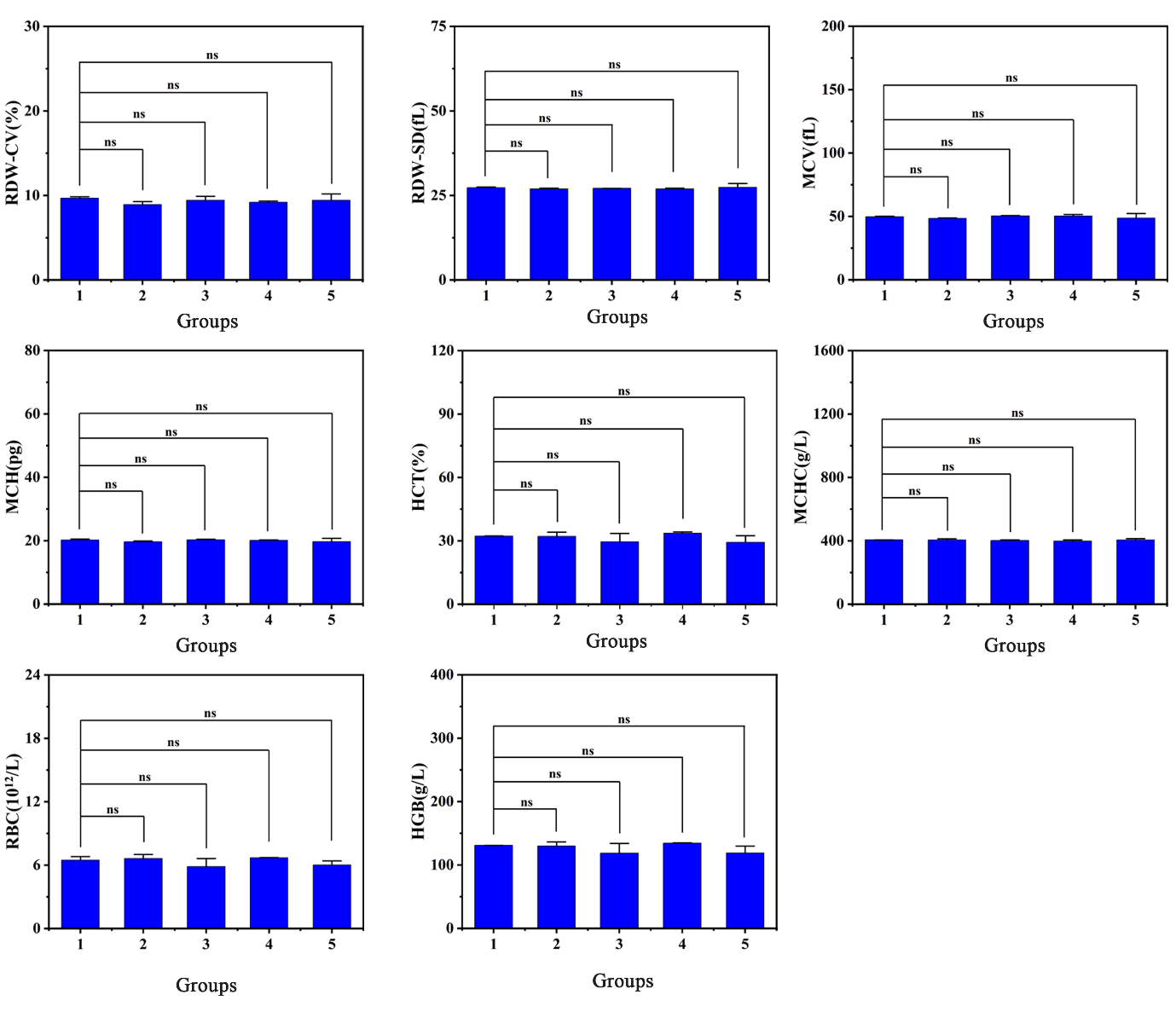


**Additonal file 1: Figure S20.** Blood routine examination results of RBC at 4 weeks. 1. Control; [2. PBNPs@OBG](mailto:2.OBG@PB); 3. OBG; 4. OBG; 5. Acupuncture (*n* = 3, ns: not significant).


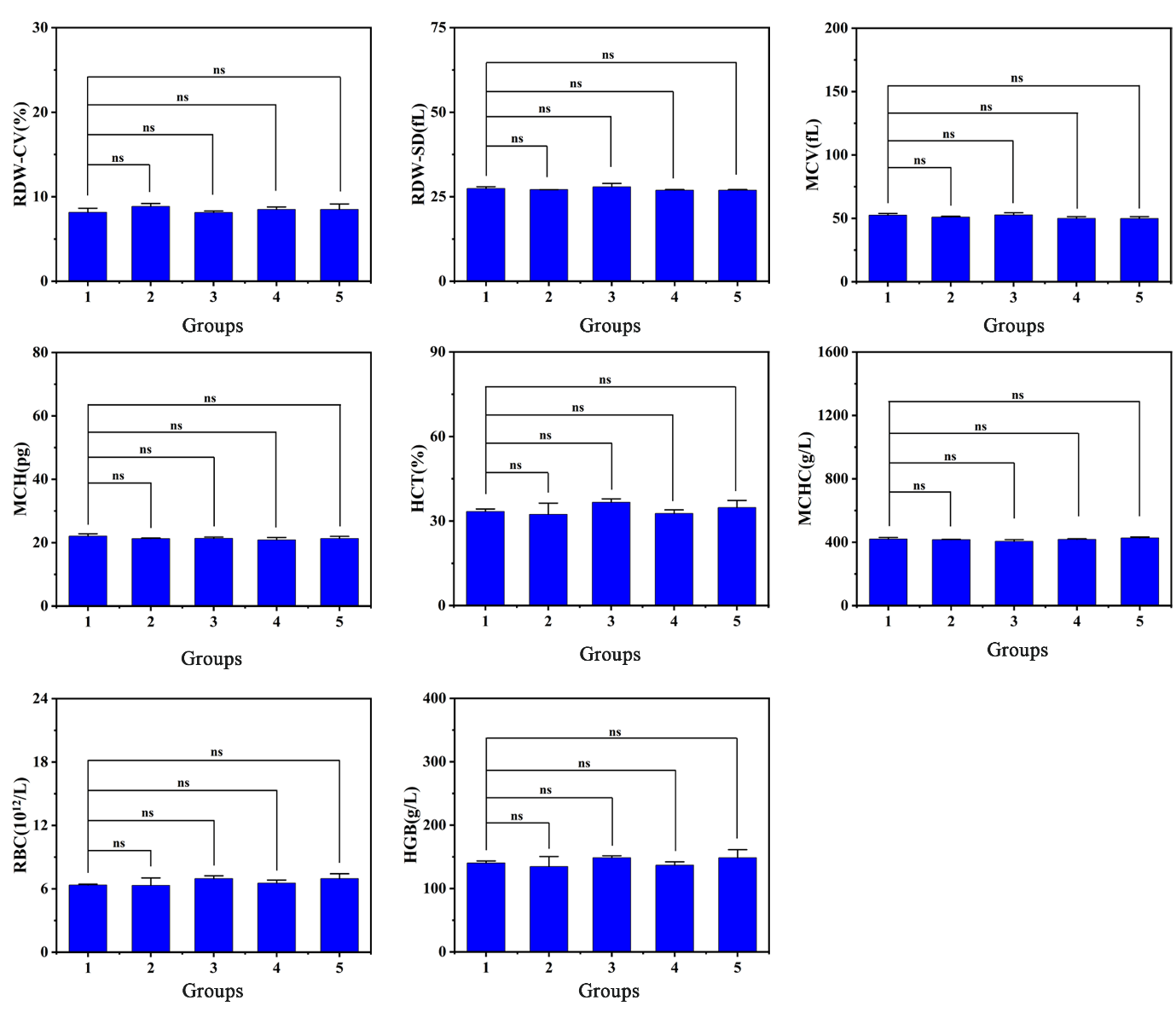


**Additonal file 1: Figure S21.** Blood routine examination results of RBC at 8 weeks. 1. Control; [2. PBNPs@OBG](mailto:2.OBG@PB); 3. OBG; 4. OBG; 5. Acupuncture (*n* = 3, ns: not significant).


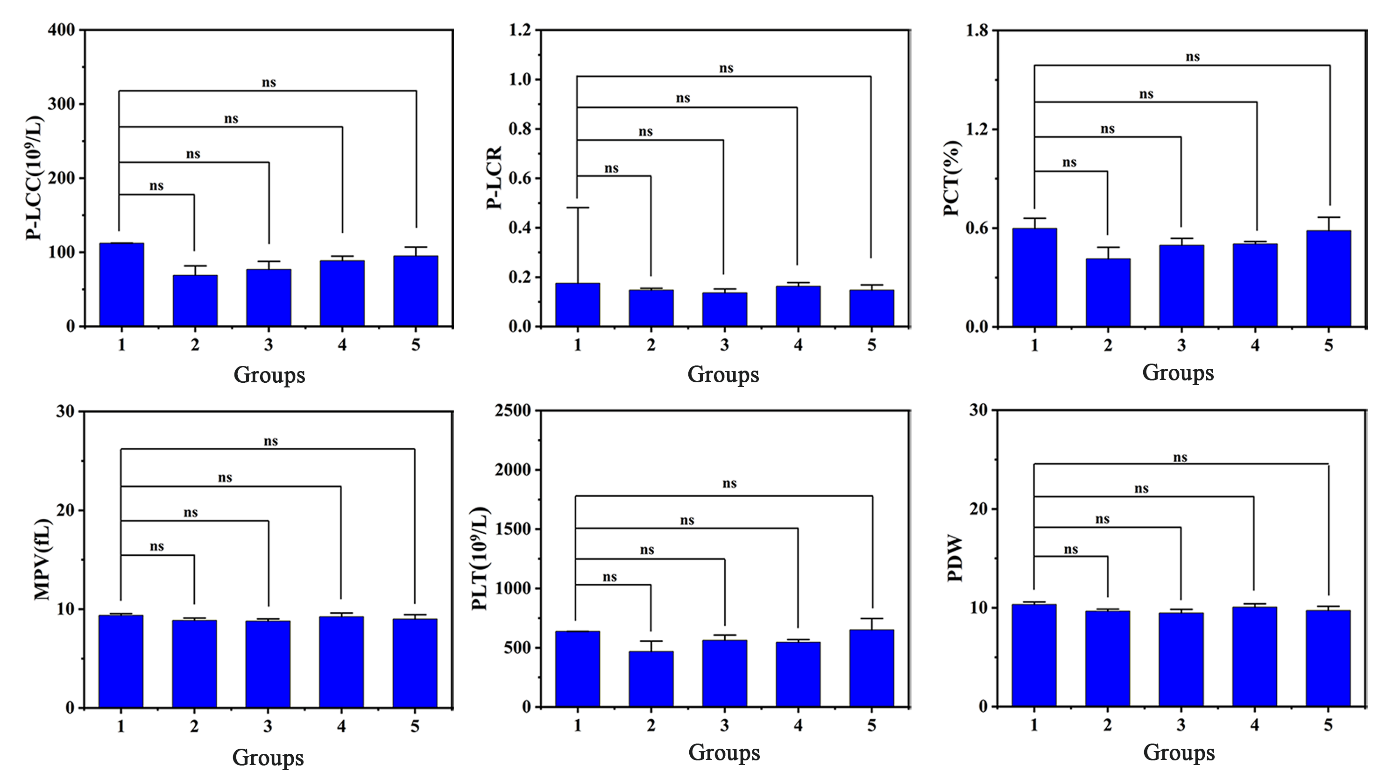


**Additonal file 1: Figure S22.** Blood routine examination results of platelets at 4 weeks. 1. Control; [2. PBNPs@OBG](mailto:2.OBG@PB); 3. OBG; 4. OBG; 5. Acupuncture (*n* = 3, ns: not significant).


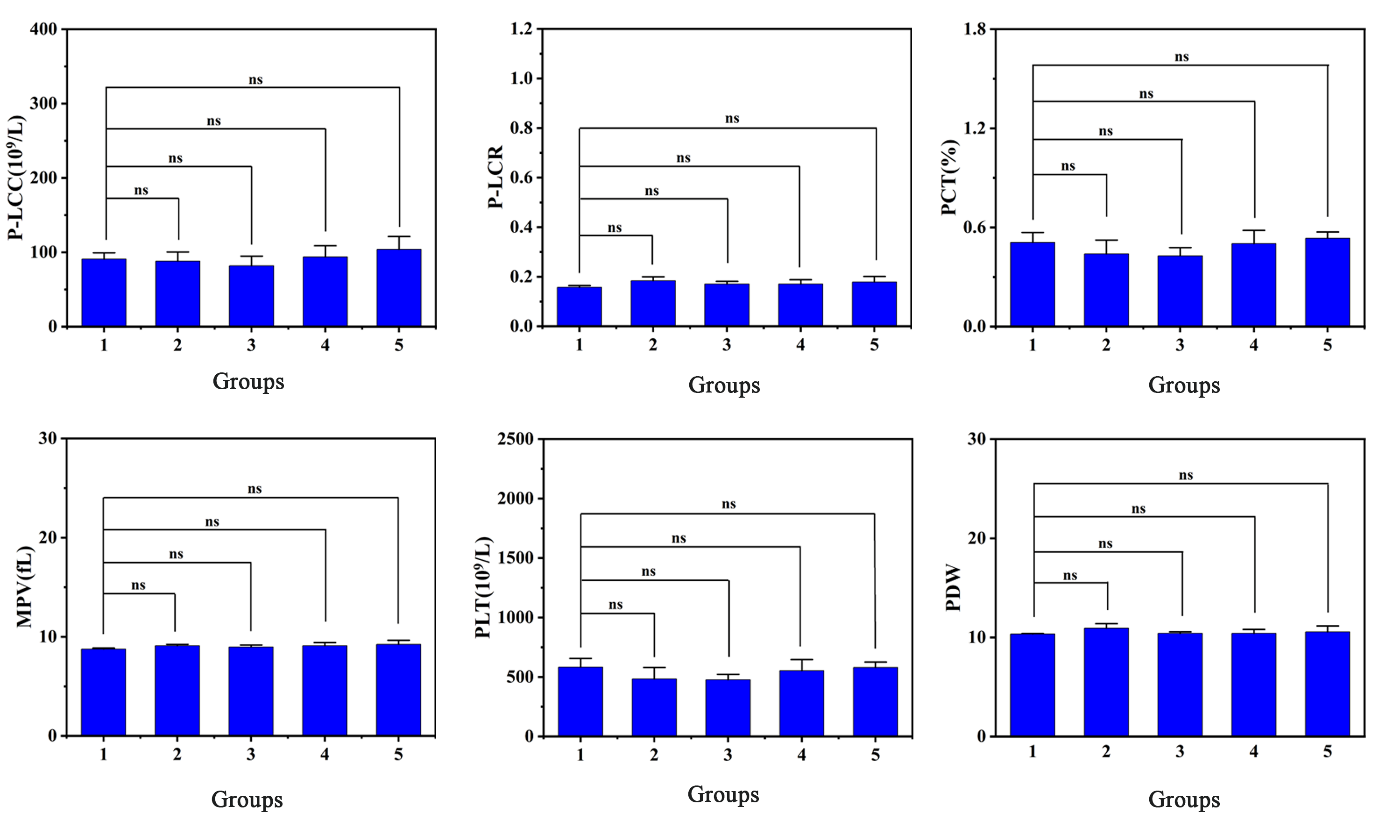


**Additonal file 1: Figure S23.** Blood routine examination results of platelets at 8 weeks. 1. Control; [2. PBNPs@OBG](mailto:2.OBG@PB); 3. OBG; 4. OBG; 5. Acupuncture (*n* = 3, ns: not significant).


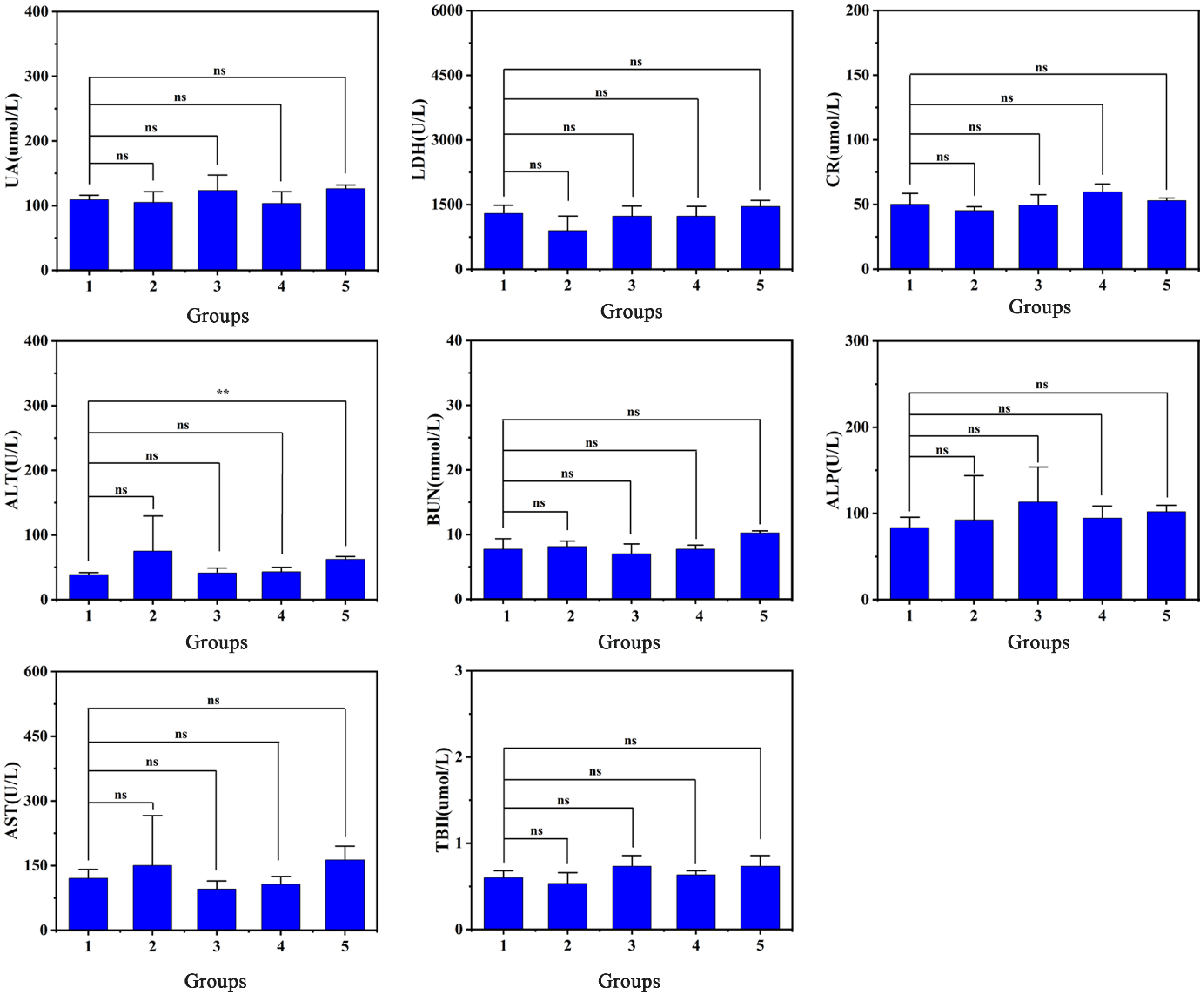


**Additonal file 1: Figure S24.** Standard blood biochemical examination at 4 weeks. 1. Control; [2. PBNPs@OBG](mailto:2.OBG@PB); 3. OBG; 4. OBG; 5. Acupuncture (*n* = 3, ***P* < 0.01, ns: not significant).

Significant differences were seen in ALT between the Control and the Acupuncture groups at 4 weeks, considering no differences were observed further at 8 weeks, it might be attributed to individual differences.


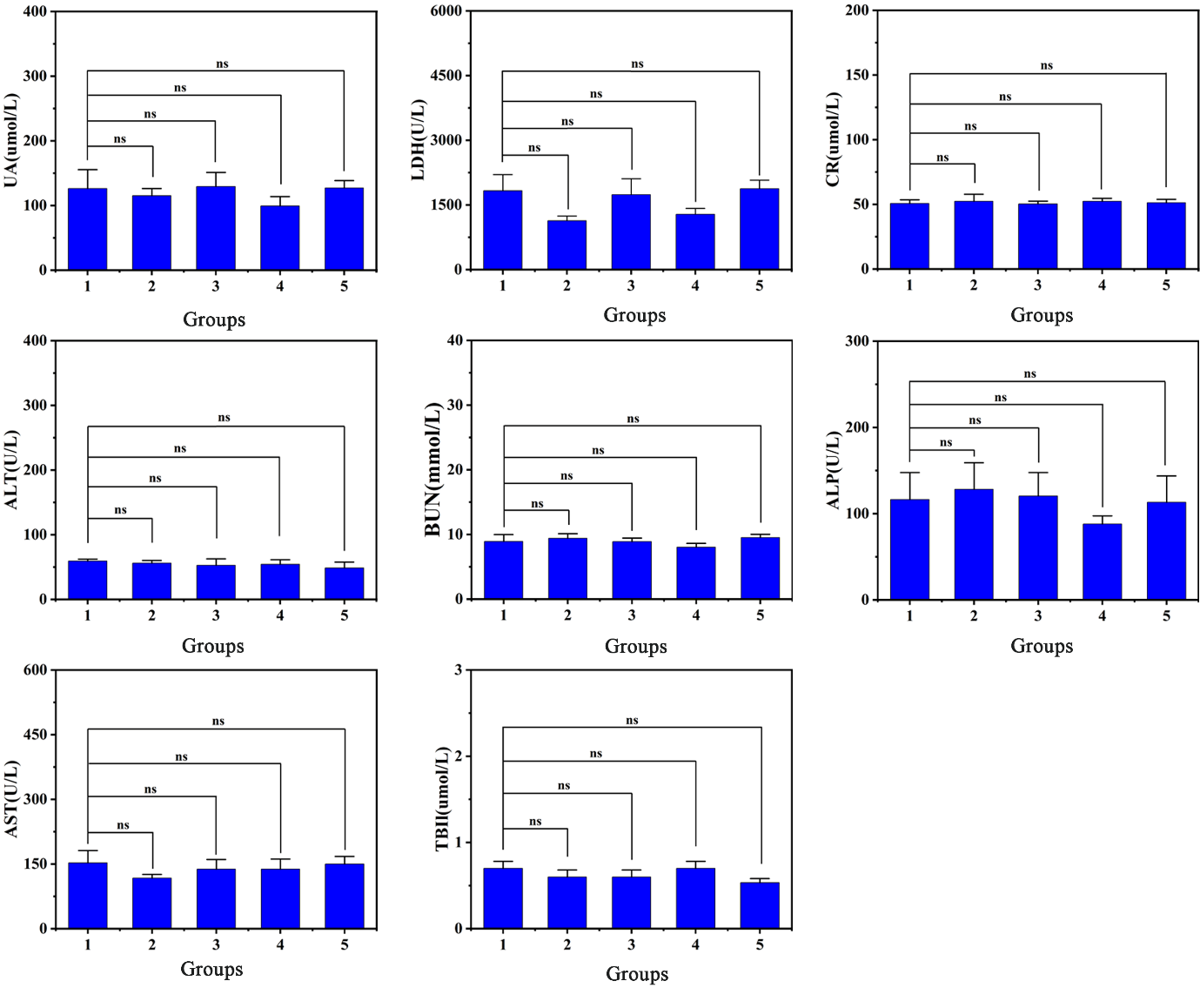


**Additonal file 1: Figure S25.** Standard blood biochemical examination at 8 weeks. 1. Control; [2. PBNPs@OBG](mailto:2.OBG@PB); 3. OBG; 4. OBG; 5. Acupuncture (*n* = 3, ns: not significant).


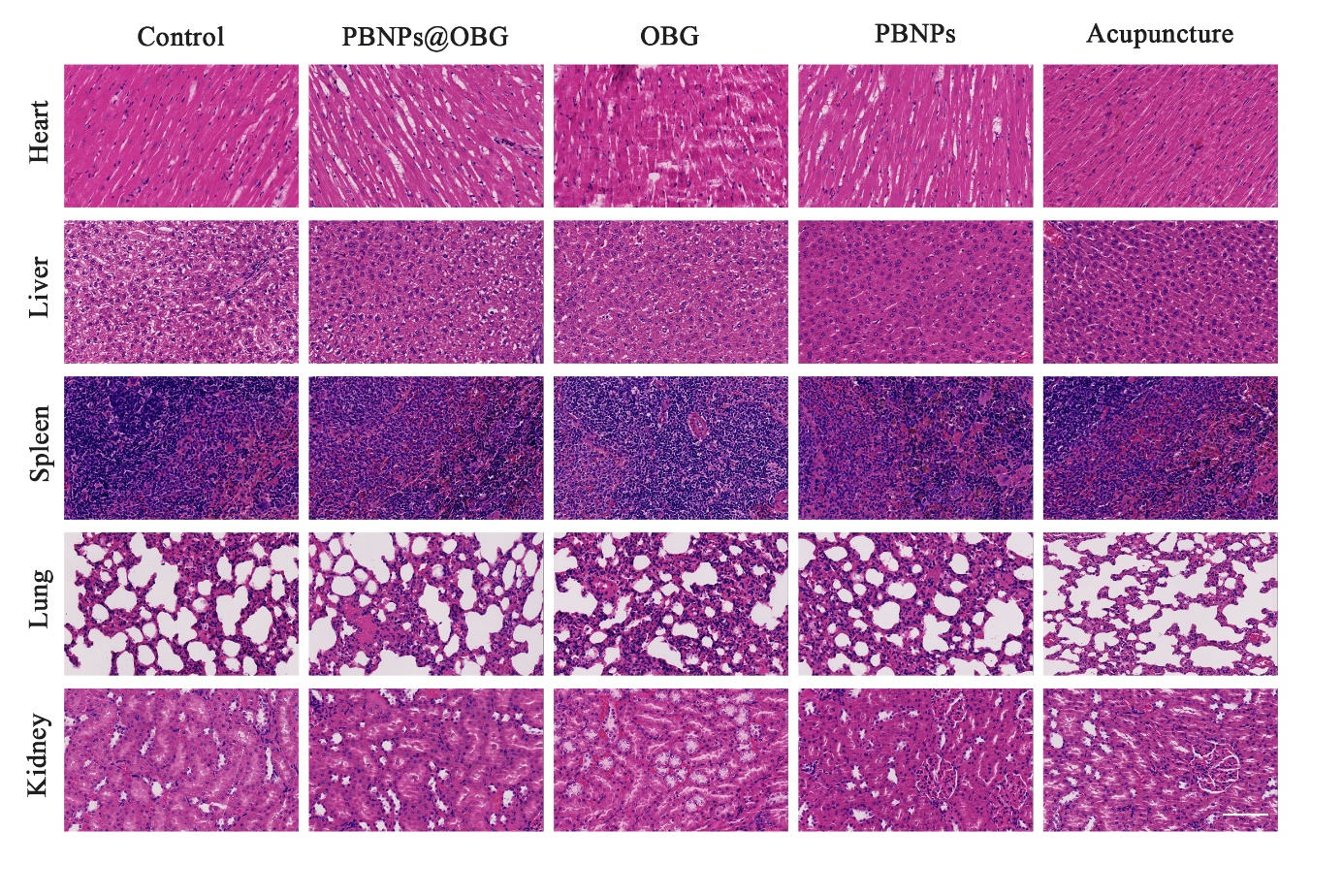


**Additonal file 1: Figure S26.** H&E staining of heart, liver, spleen, lung, and kidney at 4 weeks after operation. Scale bar = 100 µm.


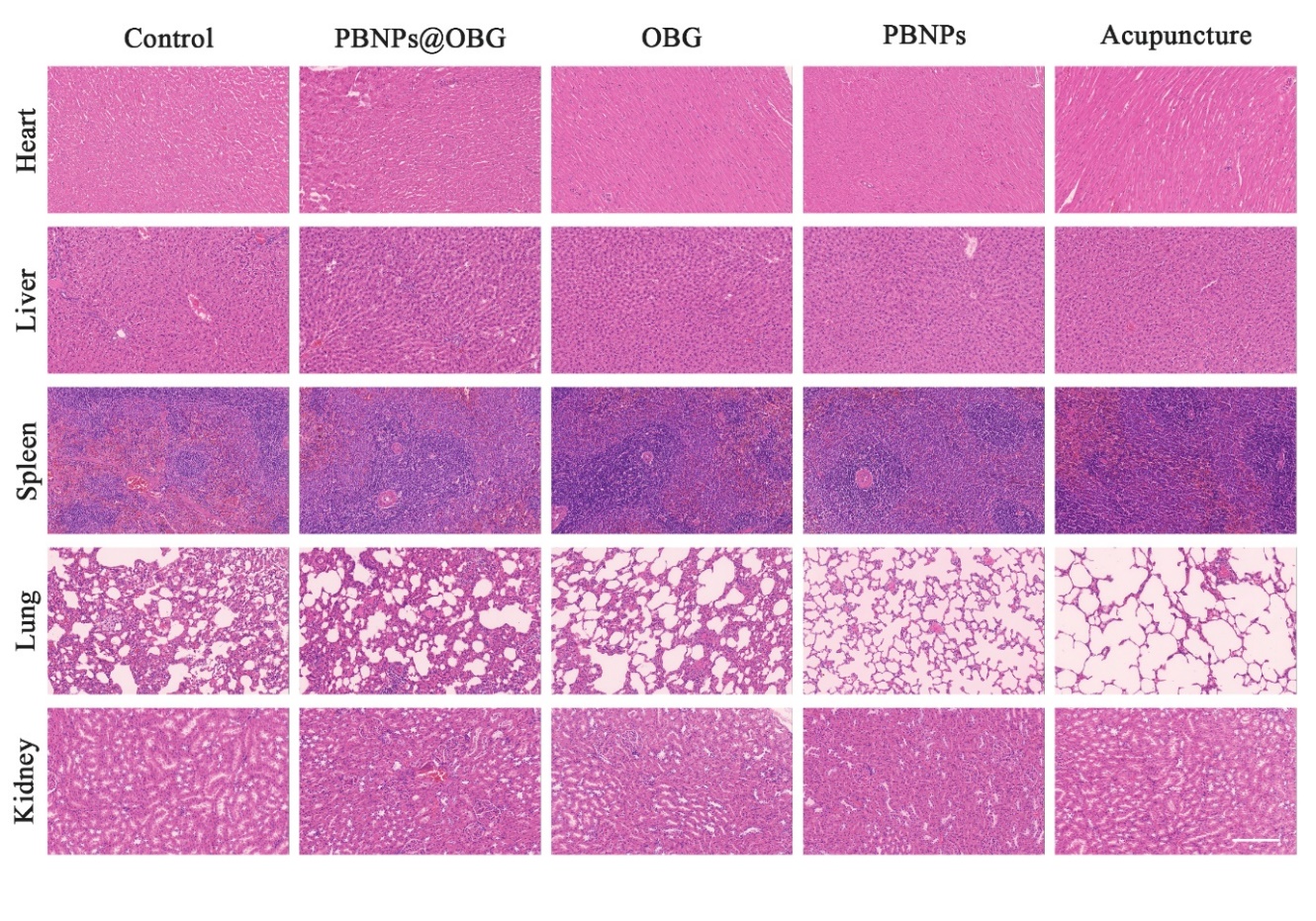


**Additonal file 1: Figure S27.** Histological screening at 8 weeks after operation. Scale bar = 100 µm.

**Additonal file 1: Table S1**. Primer used for qPCR

| Gene | Species | Direction | Sequences 5’-3’ |
| --- | --- | --- | --- |
| β-actin | Human | Forward | AGAGCTACGAGCTGCCTGAC |
|  |  | Reverse | AGCACTGTGTTGGCGTACAG |
| SOX9 | Human | Forward | CGCCATCTTCAAGGCGCTGC |
|  |  | Reverse | CCTGGGATTGCCCCGAGTGC |
| Col Ⅱ | Human | Forward | CCAGATGACCTTCCTACGCC |
|  |  | Reverse | TTCAGGGCAGTGTACGTGAAC |
| MMP3 | Human | Forward | GCTGTTTTTGAAGAATTTGGGTTC |
|  |  | Reverse | GCACAGGCAGGAGAAAACGA |
| MMP13 | Human | Forward | ATGCAGTCTTTCTTCGGCTTAG |
|  |  | Reverse | ATGCCATCGTGAAGTCTGGT |

**References**

1. A GK, B JF. Efficiency of ab-initio total energy calculations for metals and semiconductors using a plane-wave basis set. *Comp Mater Sci* 1996, 6:15-50.

2. Kresse G, Furthmüller J. Efficient iterative schemes for ab initio total-energy calculations using a plane-wave basis set. *Phys Rev B Condens Matter* 1996, 54:11169-11186.

3. Perdew JP, Burke K, Ernzerhof M. Generalized Gradient Approximation Made Simple. *Phys Rev Lett* 1996, 77:3865-3868.

4. Blöchl PE. Projector augmented-wave method. *Phys Rev B Condens Matter* 1994, 50:17953-17979.

5. Sheppard D, Xiao P, Chemelewski W, Johnson DD, Henkelman G. A generalized solid-state nudged elastic band method. *J Chem Phys* 2012, 136:074103.

6. Sheppard D, Henkelman G: Paths to which the nudged elastic band converges. *J Comput Chem* 2011, 32:1769-1771.
